# Serum Antibody Repertoire Profiling Using In Silico Antigen Screen

**DOI:** 10.1371/journal.pone.0067181

**Published:** 2013-06-27

**Authors:** Xinyue Liu, Qiang Hu, Song Liu, Luke J. Tallo, Lisa Sadzewicz, Cassandra A. Schettine, Mikhail Nikiforov, Elena N. Klyushnenkova, Yurij Ionov

**Affiliations:** 1 Institute for Genome Sciences, University of Maryland School of Medicine, Baltimore, Maryland, United States of America; 2 Department of Biostatistic and Bioinformatics, Roswell Park Cancer Institute, Buffalo, New York, United States of America; 3 Department of Cancer Genetics, Roswell Park Cancer Institute, Buffalo, New York, United States of America; 4 Department of Cell Stress Biology, Roswell Park Cancer Institute, Buffalo, New York, United States of America; 5 Department of Surgery, University of Maryland School of Medicine, Baltimore, Maryland, United States of America; Istituto Superiore di Sanità, Italy

## Abstract

Serum antibodies are valuable source of information on the health state of an organism. The profiles of serum antibody reactivity can be generated by using a high throughput sequencing of peptide-coding DNA from combinatorial random peptide phage display libraries selected for binding to serum antibodies. Here we demonstrate that the targets of immune response, which are recognized by serum antibodies directed against sequential epitopes, can be identified using the serum antibody repertoire profiles generated by high throughput sequencing. We developed an algorithm to filter the results of the protein database BLAST search for selected peptides to distinguish real antigens recognized by serum antibodies from irrelevant proteins retrieved randomly. When we used this algorithm to analyze serum antibodies from mice immunized with human protein, we were able to identify the protein used for immunizations among the top candidate antigens. When we analyzed human serum sample from the metastatic melanoma patient, the recombinant protein, corresponding to the top candidate from the list generated using the algorithm, was recognized by antibodies from metastatic melanoma serum on the western blot, thus confirming that the method can identify autoantigens recognized by serum antibodies. We demonstrated also that our unbiased method of looking at the repertoire of serum antibodies reveals quantitative information on the epitope composition of the targets of immune response. A method for deciphering information contained in the serum antibody repertoire profiles may help to identify autoantibodies that can be used for diagnosing and monitoring autoimmune diseases or malignancies.

## Introduction

The repertoires of serum antibody specificities contain information on the state of health and disease of individual. For example, circulating serum autoantibodies against self-antigens can serve as indicators of autoimmune diseases or of immune response against malignancies [1]. The information contained in the individuals’ sera can be investigated using methods for global analysis of serum antibody repertoires.

Random peptide phage display libraries (RPPDL) are widely used for mapping epitopes on defined antigens. [2]. Epitopes recognized by monoclonal as well as by polyclonal antibodies can be identified by the biopanning procedure, an affinity selection for binding to antibodies of phage displayed peptides, followed by sequencing of individual phage DNA [3], [4]. Since the length of a consensus sequence that mimics the core epitope recognized by antibody is frequently in the range from 4 to 6 amino acids [5], [6], and since all possible 6-mer amino acid permutations can be represented by 6.4×107 sequences, this implies that all possible linear core epitopes of the human proteome can be represented by the commercially available library of random heptapeptides of the complexity of approximately 109 different sequences. The necessity to sequence individual phage clones until recently limited the application of the RPPDL to identifying epitopes on a defined antigen. With the advance of next generation sequencing (NGS), the phage displayed peptides affinity selected for binding to serum antibodies can be used for generating global profiles of serum antibody specificities [7]. The feasibility of using RPPDL and NGS to analyze antibody specificities of polyclonal sera was demonstrated recently by profiling polyclonal sera derived from HIV infected individuals [8]. The authors demonstrated that a fraction of the most abundant peptides selected for binding to IgG antibodies of HIV positive sera could be aligned by a BLASTP analysis to the HIV protein thus indicating some HIV specificity.

The drawback of using RPPDL for global profiling of serum antibody reactivity is the lack of information on the identities of the real antigens that are mimicked by the antibody-binding peptides. Identifying the targets of antibody immune response using peptide mimotopes is a difficult task, since most of epitopes recognized by antibodies are conformational and discontinuous and only a small fraction of antibodies are raised against linear or continuous epitopes. Furthermore, identifying even linear epitopes of unknown targets is also complicated since a BLAST search of protein databases retrieves hundreds of proteins that have a sequence match to a short peptide. In this work we demonstrate an algorithm, which we term in Silico Antigen Screen (SAS), for analyzing peptide profiles of serum antibody specificities generated by RPPDL panning and NGS. The algorithm can be used for identifying protein autoantigens if the unknown targets are recognized by antibodies directed against linear epitopes.

## Results

### Profiling the Immune Response in Mice Immunized With Human Proteins

Peptides selected for binding to serum antibodies in biopanning experiments can mimic linear (continuous) epitopes and conformational (discontinuous) epitopes of protein antigens. They also can mimic non-protein epitopes, such as the carbohydrate structures of glycoproteins or glycolipids, nucleic acids or other chemical structures. We hypothesized that the protein targets of humoral immune response can be identified using peptide profiles of serum antibody repertoires generated by NGS, and that peptides that mimic linear eptopes of proteins will be present among the profile-making peptides., Since for any short peptide the BLAST search of protein databases retrieves about a hundred of proteins that have matches to the peptide sequence, it is practically impossible to determine what kind of epitope the peptide was mimicking. However, the BLAST search for a large number of peptides can retrieve proteins that have multiple matches to different peptides. The probability for a protein to have multiple matches to different peptides due to a chance is proportional to the length of the protein. Therefore, the real protein targets of humoral immune response recognized by antibodies directed at the linear epitopes are likely to have the higher number of matches per protein length that the proteins that have matches to peptides due to a chance.

To test this hypothesis, we used the sera of mice immunized with human proteins, prostate specific antigen (PSA) or prostatic acid phosphatase (PAP) in Complete Freund’s adjuvant. All the sera had high (>10,000) titers against the whole PAP or PSA proteins in the ELISA assay (data not shown). Our goal was to test whether it was possible to identify the proteins used for immunization by analyzing peptide profiles of serum antibody repertoires. The peptide profiles for the IgG antibodies from the four anti-PSA sera, four anti-PAP sera and two sera from naive mice were processed using two rounds of panning as described in the “Materials and Methods” section. The peptide-coding DNA fragments from the RPPDL selected for binding to the IgG antibodies were sequenced using Ilumina HiSeq 2000 and the DNA sequences were translated into the peptide sequences.

For each serum sample from mice immunized with the PSA antigen, we selected the 500 most abundant peptides that were not shared by the mice immunized with the PAP antigen or the naive mice. Excluding the peptides shared by PAP-immunized mice and by naive mice minimized the input of the response to the components of adjuvant used for immunizations and enriched the peptide list with peptides related to a specific antigen. Similarly, for each serum sample from mice immunized with the PAP antigen we selected 500 the most abundant peptides that were not shared by the PSA immunized mice or by naive mice (Table S1).

The search of the refseq_protein database for the homo sapiens (taxid:9606) using default parameters for the Blastp (protein-protein BLAST) retrieved for each peptide sequence the list of 100 proteins ranked by the decrease in the maximum score or by the increase in the expected threshold value. We tested the following 2-step algorithm for distinguishing the real antigens recognized by serum antibodies from the ‘sea’ of proteins retrieved from the database due to a chance. In the first step, we selected a limited number of the most abundant peptides and used BLAST homology search against human protein database to identify proteins that contain matches to at least two different peptides. Selecting only the most abundant peptides for this analysis would allow identifying proteins that are recognized by serum antibodies with the highest titer. Such antibodies would be easier to detect for independent confirmation of the immune response using a conventional method such as ELISA.

The number of proteins to be selected for each peptide in the first step can be regulated by varying the threshold parameters of BLAST search such as expected value (E-value) or maximal score. Lowering the E-value or increasing the maximal score allows selecting the lower number of proteins but with higher degree of homology to peptides. We chose to use the maximal score equal 18.5 as a threshold parameter, which corresponded to the match between a peptide and a protein of a stretch of 5 amino acids., For each peptide, the BLAST search retrieved, on average, approximately thirty proteins with the maximal score more than 18.5. All proteins tretrieved by the BLAST search, that satisfied the threshold parameter were combined in one list. This protein list was analyzed to select proteins which were present in the list more than once. The selected proteins were ranked by the number of matching peptides per protein length. The proteins with the highest number of matching peptides per protein length were further analyzed in the second step. In the second step, we used the Specialized BLAST tool ‘Align two (or more) sequences using BLAST (bl2seq)’ to analyze all the 500 peptides in order to identify for each selected protein all the peptides with the homologies. The less stringent threshold parameters of the bl2seq allow identifying also the peptides with lower degree of homology to proteins, which could be missed in the first step of the algorithm. The candidate targets of immune recognition were selected by ranking according to the final score calculated for each selected protein as the sum of scores for all the peptides with the homology to the protein divided by the length of the protein.

When we applied this algorithm to the lists of 500 peptides for the anti-PSA sera, and used a BLAST search against human refseq_protein database (first step), we found that none of the peptides retrieved the PSA protein. This may indicate that the humoral response to the human PSA protein in mice is limited to conformational epitopes. In contrast, the analysis of PAP-specific sera produced multiple hits. Three anti-PAP sera samples (PAP1, PAP2 and PAP3) produced the peptide lists with multiple peptides that retrieved the PAP protein by BLAST search against human refseq_protein database. One anti-PAP serum (PAP4) produced the 500 peptide list that contained only one peptide which retrieved the PAP protein by BLAST search against human refseq_protein database in the first step.

The first 120 most abundant peptides of the PAP1 list contained 2 peptides that retrieved the PAP protein with the maximal score ≥18.5. Besides the 2 isoforms of the PAP proteins NP_001090.2 and NP_001127666.1, there were 194 other proteins that had multiple matches to different peptides. After ranking the proteins according to the initial score calculated as a number of matching peptides to the protein divided by the length of the protein, the PAP isoforms NP_001090.2 and NP_001127666.1 occupied the 56th and 66th positions respectively (Table S2A).

In the second step of the algorithm, we run the bl2seq for the all 500 peptides of the PAP1 list against each of the first top 100 proteins with multiple matches identified in the first step. For this purpose, we wrote software that allows performing bl2seq off line for the large number of peptides against large number of proteins. These analyses identified all the peptides in the list with matches to selected proteins. The proteins were then ranked according to the final score that was calculated as the sum of the scores for the all peptides with matches against the protein divided by the length of the protein. Such analyses ranked the PAP isoforms NP_001090.2 and NP_001127666.1 at the 4th and the 7th positions, respectively (Table S2A and Table 1).

**Table 1:** Candidate antigens for mouse sera.

Rank	Proteins selected for PAP1 antiserum	Protein length(aa)	Initial number of matches	Initial score	Sum of overall scores	Final score	Major epitope score
1	NP_065117.1 claudin-2	230	2	0.0086	600.1	2.60913	147.9
2	XP_003119043.1 PREDICTED: hypothetical protein LOC100506191	118	2	0.0169	266.2	2.255932	126.2
3	NP_653235.1 lysozyme-like protein 4 precursor	146	2	0.0136	302.4	2.071233	163
4	*NP_001090.2 prostatic acid phosphatase isoform PAP precursor*	*386*	*2*	*0.0051*	*769.5*	*1.993523*	*376*
5	NP_001181944.1 T-cell surface glycoprotein CD4 isoform	185	2	0.0108	361.9	1.956216	218.5
6	NP_001098018.1 uncharacterized protein	102	3	0.0294	192.9	1.891176	76.3
7	*NP_001127666.1 prostatic acid phosphatase isoform TM-PAP precursor*	*418*	*2*	*0.0047*	*777.3*	*1.859569*	*376*
8	NP_057574.2 protein FAM178B isoform B	119	2	0.0168	210.2	1.766387	0
9	NP_001172078.1 putative claudin-24	220	2	0.009	369.4	1.679091	0
10	NP_000139.1 galactoside 2-alpha-L-fucosyltransferase 1	365	2	0.0054	594	1.627397	0
	**Proteins selected for PAP2 antiserum**						
1	NP_001230678.1 modulator of retrovirus infection homolog isoform 2	69	5	0.0724	1260.4	18.26667	1245
2	NP_001011700.2 mitochondrial coiled-coil domain protein 1 precursor	119	3	0.0252	1414.3	11.88487	1350.5
3	NP_001092926.2 neuropeptide W preproprotein	165	2	0.0121	1425	8.636364	1281.6
4	NP_076938.2 modulator of retrovirus infection homolog isoform 1	157	5	0.0318	1293.6	8.23949	1245
5	NP_000396.2 ganglioside GM2 activator isoform 1 precursor	193	2	0.0103	1461.6	7.573057	1236.7
6	NP_065117.1 claudin2	230	2	0.0086	1658.4	7.210435	1154.9
7	NP_997394.1 uncharacterized protein C9orf139	190	3	0.0157	1290.5	6.792105	984
8	NP_114103.2 voltage-dependent calcium channel gamma-6 subunit isoform c	189	3	0.0158	1262.2	6.678307	1157.5
9	NP_942567.1 probable alpha-ketoglutarate-dependent dioxygenase ABH6 isoform 1	161	2	0.0124	1063.8	6.607453	989.5
10	NP_443183.1 deoxynucleotidyl-transferase terminal-interacting protein 1	329	9	0.0273	2045.1	6.216109	1866.1
11	NP_665814.1 voltage-dependent calcium channel gamma-6 subunit isoform b	214	3	0.014	1277.4	5.969159	1157.5
12	*NP_001090.2 prostatic acid phosphatase isoform PAP precursor*	*386*	*7*	*0.0181*	*2245.2*	*5.81658*	*1886.9*
13	NP_005182.1 T-lymphocyte activation antigen CD80 precursor	288	2	0.0069	1666.3	5.785764	1415.9
14	NP_001010905.1 UPF0762 protein C6orf58 precursor	330	3	0.009	1781.4	5.398182	1276.3
15	*NP_001127666.1 prostatic acid phosphatase isoform TM-PAP precursor*	*418*	*7*	*0.0167*	*2245.2*	*5.371292*	*1886.9*
	**Proteins selected for PAP3 antiserum**						
1	NP_149101.1 gamma-glutamylaminecyclotransferase	153	5	0.0326	1054.8	6.894118	930.3
2	NP_001165160.1 AMME syndrome candidate gene 1 protein isoform 3	210	4	0.019	1032.3	4.915714	897.8
3	NP_001025046.1 regulator of G-protein signaling 7-binding protein	257	5	0.0194	1254.4	4.880934	1043.1
4	NP_060436.4 vacuolar protein sorting-associated protein 37C	355	2	0.0056	1502.4	4.232113	835.1
5	NP_113633.2 AMMECR1-like protein	310	2	0.0064	1053.7	3.399032	876.9
6	NP_003921.2 src kinase-associated phosphoprotein 2	359	2	0.0055	1207.6	3.363788	949.8
7	NP_004995.1 NADH dehydrogenase [ubiquinone] 1 beta subcomplex subunit 8, mitochondrial precursor	186	3	0.0161	618.1	3.323118	243.2
8	*NP_001090.2 prostatic acid phosphatase isoform PAP*	*386*	*6*	*0.0155*	*1159.5*	*3.003886*	*906.5*
9	NP_001504.2 maleylacetoacetate isomerase isoform 3	161	3	0.0186	481.7	2.991925	374
10	NP_005986.2 T-box transcription factor TBX10	385	2	0.0051	1127.3	2.928052	857.6
11	NP_078870.1 pantothenate kinase	370	3	0.0081	1044.1	2.821892	876.9
12	*NP_001127666.1 prostatic acid phosphatase isoform TM-PAP precurso*	*418*	*6*	*0.0143*	*1169.2*	*2.797129*	*906.5*
13	NP_665878.2 maleylacetoacetate isomerase isoform 2	174	3	0.0172	478.3	2.748851	374
14	NP_001180318.1 putative E3 ubiquitin-protein ligase UNKL isoform	182	3	0.0164	476.2	2.616484	0
15	NP_061867.1 F-box only protein 42	717	4	0.0055	1764.7	2.461227	1024.7

The table shows the top candidate antigens selected for the antibodies of the PAP1, PAP2 and PAP3 antisera by sorting the data generated by processing the results of BLAST database search for the corresponding peptide sequences.

**Figure 1:**
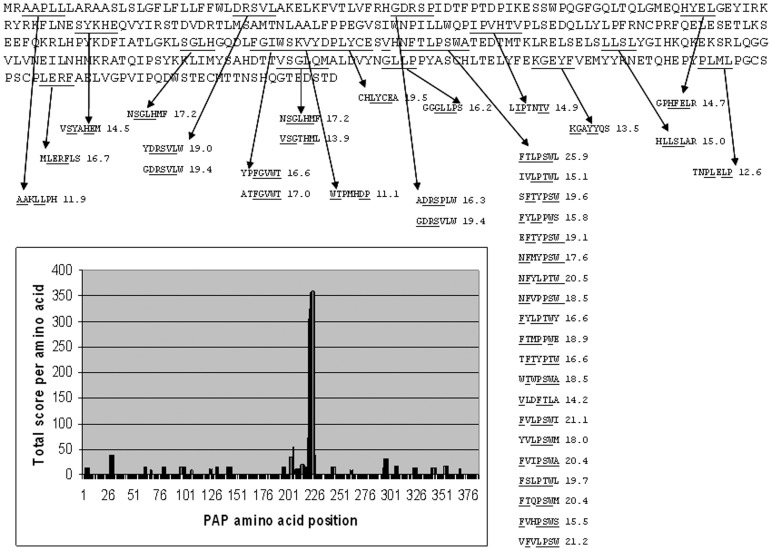
Epitope profile of the PAP protein generated by PAP1 mouse antiserum. (**A**) The NP_001090.2 PAP variant amino acid sequence and the 40 matching peptides with the highest match scores generated by bl2seq program are shown. The sequences of the protein matching to peptides and the corresponding peptide sequences that match to protein are underlined. **B**. The graphic shows the distribution of the total score of the peptide matching over the sequence of the PAP protein.

The Blast2seq analysis for the 500 PAP1 peptides against PAP NP_001090.2 or NP_001127666.1 isoforms identified 48 peptides with matches to the PAP protein. Out of 48 peptides, 20 were related to the sequence NFTLPSWA located at the amino acid positions 220–227 of the PAP protein. Figure 1 shows the location of peptide matches on the sequence of the NP_001090.2 PAP variant. Three other proteins, that were ranked higher than the NP_001090.2 PAP isoform also contained the sequences related to the NFTLPSWA sequence and had multiple matches to related peptides. However, the sum of scores for the peptides related to the NFTLPSWA sequence of the PAP isoforms was the highest among the sums of scores for the related epitopes in other proteins (Table 1).

The list of top 500 candidate peptides for the PAP2 serum was significantly enriched with peptides with homology to the PAP proteins sequence. The BLAST search against human refseq_protein for the first most abundant 120 peptides of the PAP2 list contained 7 peptides that retrieved the PAP protein isoforms with the maximal score ≥18.5. Out of 500 peptides of the PAP2 list analyzed by the blast2seq, 136 peptides matched the PAP isoforms, of which 110 peptides were related to the NFTLPSWA sequence, which was the same sequence related to the peptides for the PAP1 sample. Besides 2 isoforms of the PAP protein, there were 490 other proteins that had multiple matches to different peptides. After ranking the top 100 proteins identified in the first step of analysis using the blast2seq, the PAP isoforms occupied the 12th and the 15th positions (Table S2B and Table 1). The proteins that were ranked higher than the PAP isoforms all had the sequences related to the NFTLPSWA sequence. The higher final scores of these proteins were caused by the input from peptides that have matches to these sequences. As was the case for the PAP1 serum, the sum of scores for the PAP2 peptides related to the NFTLPSWA sequence of the PAP isoforms was higher than the sum of scores for the peptides that have matches to the related sequences in other proteins.

The BLAST search of the first 120 peptides of the PAP3 500 peptide list also contained PAP isoforms as well as 444 proteins that had multiple matches to different peptides with the threshold maximal score ≥18.5. After ranking the top 100 proteins identified in the first step of analysis using the blast2seq, the PAP isoforms occupied the 8th and the 12th positions (Table S2C and Table 1). Out of 500 peptides of the PAP3 list, the 69 and 70 peptides had the matches to the two respective PAP isoforms, of which 48 peptides were related to the amino acids 336–342 QHEPYPL sequence of the PAP protein. The proteins that were ranked higher than the longest NP_001127666.1 PAP isoform also contained the motifs related to the QHEPYPL sequence. The sum of the scores for the QHEPYPL epitope of the PAP isoforms was ranked as 5th among the similar epitopes in other proteins. Validating the SAS Results of Mouse Sera Profiling by Bioinformatics Analyses.

**Figure 2:**
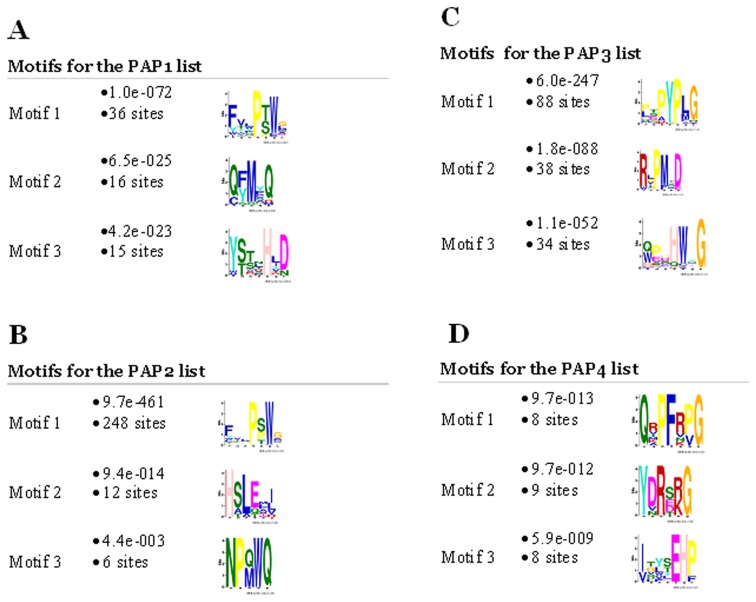
Motifs identified by MEME software for the 500 peptide lists for the PAP1, PAP, PAP3 and PAP4 antisera.

The BLAST-based analysis of serum antibody repertoire described above can identify the linear epitopes but will miss mimotopes that mimic the non-linear protein or carbohydrate epitopes. To identify all the motifs recognized by serum antibodies that represent linear and non-linear epitopes we analyzed the lists of peptides using MEME software available online http://meme.sdsc.edu/meme/cgi-bin/meme.cgi. Figure 2 shows the three motifs identified by MEME for the each PAP antiserum. For the PAP1, PAP2 and PAP3 antisera, the most represented motifs were related to the NFTLPSWA and QHEPYPL sequences of the PAP protein. For the PAP4 serum that did not produce significant matches to the PAP protein by BLAST analysis, all three motifs were represented equally.

**Figure 3:**
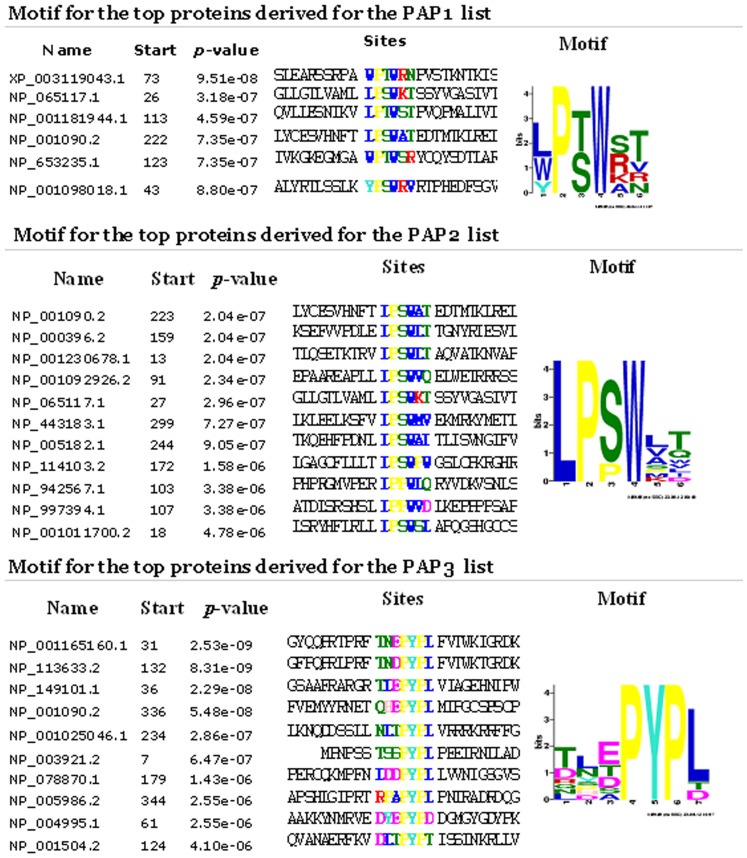
Motifs identified by MEME software for the top candidate antigens selected for the PAP1 and PAP3 antisera. Motifs for the antigens are related to the motifs identified for the corresponding top 500 the most abundant peptides.

We also used MEME software to analyze the sequences of proteins that had been selected as the candidate antigens for the PAP1, PAP2 and the PAP3 sera based on their higher final score compared to the PAP isoforms. The MEME analysis identified the same motifs related to the NFTLPSWA and the QHEPYPL sequences of the PAP protein (Figure 3), suggesting that the PAP1, PAP2 and PAP3 sera could cross-react with these proteins.

We also analyzed the PAP protein sequence using available online tool for linear epitope prediction http://sysbio.unl.edu/SVMTriP/prediction.php. The software based on the Support Vector Machine algorithm predicted existence of three linear epitopes within the PAP sequence (Table 2). Although the NFTLPSWA sequence was not included in any of the predicted epitopes, the epitope predicted with the highest score included the QHEPYPL sequence recognized by the PAP3 antiserum. Another predicted epitope contained the match to the peptide NTTNSHG from the PAP3 list, which retrieved the PAP isoforms by the BLAST searching of the peptide sequence against human refseq_protein database.

**Table 2:** Epitopes predicted by the SVMTriP inside the PAP protein.

Rank	Location	Epitope	Score
1	334–353	ET**QHEPYPL**MLPGCSPSCPL	2.505
2	136–155	LLWQPIPVHTVPLSEDQLLY	1.152
3	360–379	VGPVIPQDWSTECM**TTNSH**Q	1.004

The table shows the hypothetical epitopes predicted by the SVMTriP software on the PAP protein. In bold are the sequences that produce matches to the peptides recognized by serum antibodies of the Pap3 serum.

### Validating the SAS Results of Mouse Sera Profiling Using the Anti-peptide ELISA

**Figure 4:**
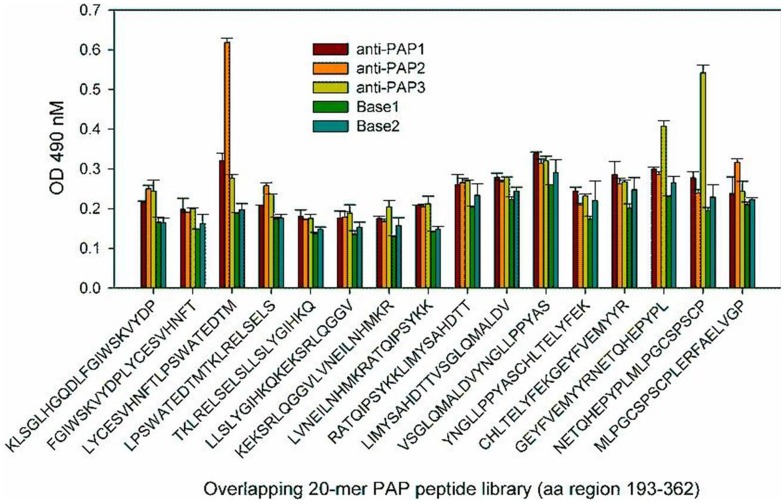
Epitope mapping for human PAP-specific antibodies from four immune sera samples The reactivity of 4 mouse PAP-specific immune sera (PAP1, PAP2, PAP3 and PAP4) was tested by ELISA using a library of overlapping 20-mer peptides that span the entire amino acid sequence of human PAP. Base1 and base 2 samples derived from naïve mice served as negative controls. The results for the PAP protein region corresponding to amino acids 193–362 are shown.

To prove that the sequences identified by the SAS method represent the real linear epitopes recognized by serum antibodies, we analyzed PAP-specific antisera by ELISA using peptide library consisting of 20-mers that overlap by 10 amino acids and span the mature human PAP amino acid sequence. As shown in Figure 4, PAP1 and PAP2 antisera recognized 20-mer peptide containing the NFTLPSWA sequence, and PAP3 antiserum recognized the 20-mer peptides containing the QHEPYPL sequence. The analysis of PSA-specific antisera by ELISA using the overlapping peptides representing the PSA proteins did not identify any peptide that had a signal significantly higher than that for the background binding (not shown) thus confirming the lack of recognition of linear epitopes of the PSA in the analyzed PSA-specific antisera.

### Analyzing Antibody Repertoire of Human Serum

**Figure 5:**
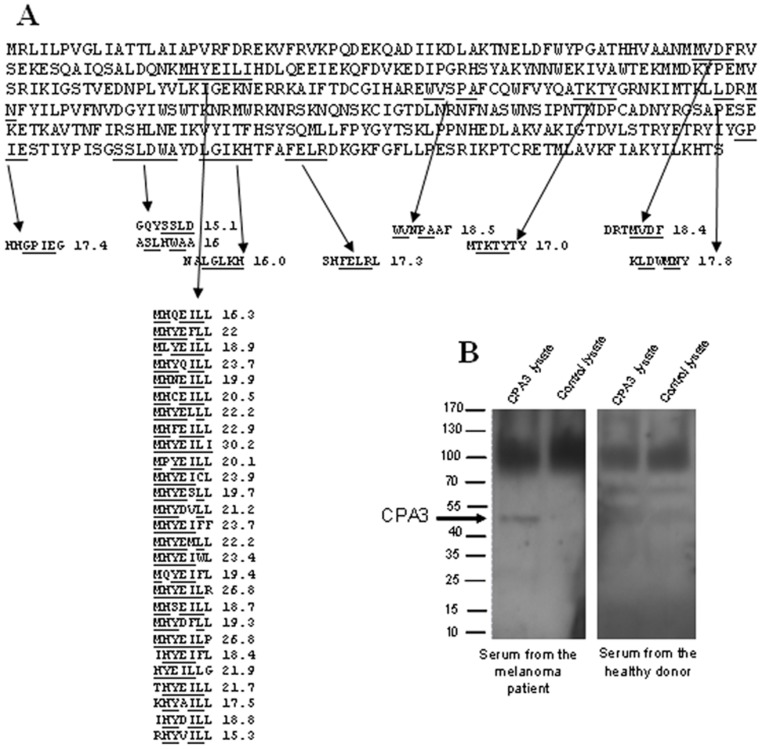
Epitope profile of the CPA3 protein generated by the serum from a melanoma patient. (**A**). The CPA3 amino acid sequence and the 36 matching peptides with the highest match scores generated by bl2seq program are shown. The sequences of the protein matching to peptides and the corresponding peptide sequences that match to protein are underlined. (**B**). Western blot analysis of the 293T cell lysates overexpressing the recombinant CPA3 protein or transfected with empty vector. The membranes were incubated with serum from either melanoma patient (left panel) or healthy donor (right panel). The reaction was developed as described in the “Materials and Methods” section.

The described analysis of mouse sera using SAS demonstrates that the method can identify the antigen used for immunization, when the immune response involves recognition by serum antibodies of linear epitopes of the antigen. Next we wanted to evaluate the capability of the method to identify autoantigens recognized by serum antibodies produced in the absence of immunization. We analyzed a serum sample from the metastatic melanoma patient, assuming that the serum of a cancer patient can contain autoantibodies against proteins which are overexpressed or aberrantly expressed in tumor cells and had been exposed to the immune system due to tumor cell death. For the serum antibodies of the melanoma patient we identified the 500 most abundant peptides which were not shared with the list of peptides corresponding to the serum sample from a healthy donor. To identify the candidate autoantigens recognized by serum antibodies of the melanoma patients we used the same algorithm as we did for identifying the antigen used for immunization of mice. Table 3 shows the top 10 proteins ranked according to the final score calculated using the bl2seq program for the 500 most abundant peptides. The protein Mast Cell Carboxypeptidase A Precursor (CPA3), which had the highest final score, produced matches to the 52 peptides with the sum of scores equal 917.3. Of these peptides more than half (27) were aligned against a single site with the cumulative score equal 575.4 (Figure5). This score was comparable to the scores corresponding to the major epitopes recognized by mouse sera on the PAP molecule. To test if the serum antibodies of the melanoma patient will recognize the whole CPA3 protein we analyzed the 293T cells lysate (purchased from the origene.com) overexpressing the recombinant CPA3 protein using Western blotting. As shown in Figure 5, serum from the melanoma patient but not from the healthy donor reacted with a protein band corresponding to the size of the CPA3 protein in the 293T cells lysate overexpressing the recombinant CPA3 protein. No reactivity was detected in the control 293T cells lysate.

**Table 3:** Top candidate antigens selected by the SAS for the melanoma patient serum.

Rank	Proteins selected for PAP1 antiserum	Proteinlength(aa)	Sum of overallscores	Finalscore	Major epitopescore
1	NP_001861.2 mast cell carboxypeptidase A precursor	417	917.3	2.19976	517
2	NP_060293.2 dual specificity protein phosphatase 23	150	305.6	2.037333	252.2
3	NP_055150.1 EP300-interacting inhibitor of differentiation 1	187	318.2	1.701604	262.7
4	NP_001535.1 intercellular adhesion molecule 4 isoform 1 precursor	271	454.8	1.678229	none
5	NP_037441.2 zinc transporter 4	429	711	1.657343	280.6
6	NP_071772.1 intercellular adhesion molecule 4 isoform 2 precursor	237	385.5	1.626582	none
7	NP_001093391.1 melanoma-associated antigen B16	324	515.7	1.591667	145.4
8	NP_064576.1 28S ribosomal protein S22, mitochondrial	360	572.9	1.591389	119.7
9	NP_001034221.1 intercellular adhesion molecule 4 isoform 3 precursor	272	432.7	1.590809	none
10	NP_000307.1 parathyroid hormone-related peptide receptor precursor	593	935	1.576728	328.6

The table shows the top candidate antigens selected for the antibodies of the melanoma patient serum by sorting the data generated by processing the results of BLAST database search for the corresponding peptide sequences.

## Discussion

We have demonstrated that the lists of peptide sequences generated by the NGS of DNA from RPPDL selected for binding to serum antibodies can be used for identifying protein antigens recognized by serum antibodies, when the humoral immune response is at least partially directed to linear epitopes. The simple BLAST-based algorithm of the SAS strategy generated a list of candidate proteins that contained multiple sequence matches to different peptides from the list. The PAP protein used for immunization of mice was one of the top fifteen candidates for the three out of four immunized mice. It is noteworthy that the proteins that were ranked higher than the PAP protein shared the same motif with the PAP, suggesting that antibodies against the PAP are likely to cross-react with the identified candidate proteins. Moreover, for the two out of three sera that showed a response to linear epitopes of the PAP protein, the score for the PAP epitope was the highest among the similar epitopes in other proteins thus making the PAP protein the top candidate for the target of immune response.

In addition to identifying the candidate proteins recognized by antibodies, the SAS also exposed quantitatively the “fine structure” of humoral immune response against the antigen. SAS not only can identify the epitopes recognized by antibodies on the protein sequence but also can show the strength of the response against these epitopes, since the number of peptides containing the epitope sequence should be proportional to the titer of antibodies.

The antibody responses against the linear epitopes of the human PAP protein appear to be limited in mice only to few epitopes. Each of the PAP1, PAP2 and PAP3 antisera recognized only a single major linear epitope; for the PAP1 and PAP2 antisera, the same epitope was recognized. This result is not in contradiction with the previously observed fraction of antibodies that recognized linear epitopes. It was shown that less than 2% of the antibodies raised against the cytochrome c could also recognize the synthetic peptides. The authors concluded that linear epitopes were rare in the case of small, globular, and conformationally stable proteins. The inability of the method to identify the PSA protein which was also used for immunization can be explained by the absence of the response against the linear epitopes of the PSA protein, which is shorter than the PAP protein. We think that if the immune response in mice were to be directed against 2 or more major linear epitopes of the PAP, the PAP protein would be ranked first with a big lead over the other proteins, which were ranked higher mainly due to a shorter size than that of the PAP protein. Although the SAS has been designed for identifying proteins with multiple epitopes recognized by serum antibodies, it appears that a large number of different peptides that have matches to a single epitope may be sufficient for identifying a protein as a candidate antigen. However, when only a single major epitope is detected on the candidate protein, the possibility that the protein is identified due to a chance can not be excluded. Based on the analysis of the PAP3 serum sample we had to choose another protein, and not the PAP protein as the first candidate for the target of the immune response (Table 1). Even when binding of a protein to serum antibodies is confirmed by Western blot, as was the case with the CPA3 protein and the melanoma patient’s serum antibodies, this binding does not necessary mean that the immune response was directed against this protein, but could be attributed to the cross reactivity. It is possible that the peptide sequence recognized by serum antibodies of the melanoma patient on the CPA3 protein is the mimic of a conformational or carbohydrate epitope on the other human, viral or bacterial protein, which may represent the unknown real target of the humoral immune response. This limitation of the SAS method is shared with the other methods of serum antibody repertoire profiling which are based on using the cDNA expression libraries, such as SEREX [9].

When we analyzed the reactivity of the melanoma serum sample against the next ranked highest after the CPA3 protein, the serum antibody did not detect the corresponding band on the western blot. The absence of the specific signal for the protein on the western blot does not necessary mean the absence of autoantibodies against this protein. It can be explained by the much higher sensitivity of the SAS over the Western blot. The detection of the immunoreactivity in the SAS is based on sequencing of the PCR amplified DNA fragment coding for the peptide that binds to antibodies. The method can detect even a single peptide that binds to a single molecule of the antibody. Such sensitivity cannot be achieved by Western blot analysis or ELISA. The extremely high sensitivity of the SAS capable of identifying low affinity or low titer autoantibodies against tumor associated antigens is of importance for development of the diagnostic assays for early detection of cancer.

The efficiency of having multiple epitopes for identifying disease-relevant targets of immune response has been demonstrated previously by searching protein databases for homology against predicted epitopes of two monoclonal antibodies recognizing the same protein [5]. In this work, we demonstrated that combining RPPDL panning with high throughput sequencing allows the generation of global profiles of serum antibody repertoires. We further demonstrated that, using the BLAST algorithm to analyze a large number of peptides against human protein databases, the targets of serum antibodies can be identified without prior knowledge about the proteins that induced immune response. We suggest that the SAS method can be used for identifying novel tumor associated antigens of cancer patients as well as the targets of immune response in autoimmune diseases. The identified by the SAS tumor-associated antigens can be used for developing personalized immunoassays for detecting recurrences and for personalized anticancer immunotherapies. The applicability of the SAS for identifying antigens beyond human proteome needs to be tested. It is unlikely that the unknown pathogen can be identified by performing BLAST search for short peptides against the non-reduntant protein sequences database that includes more than 30 millions of proteins from more than 20 thousands organisms. However, when the source of a pathogen is known to be associated with viruses or bacteria the BLAST search against bacterial or viral protein database is likely to be capable of identifying proteins containing linear epitopes.

Although the SAS is designed for identifying linear epitopes, a high throughput sequencing of random peptide phage libraries selected for the large number of serum samples from infected individuals can identify peptide motifs associated with viral or bacterial infections. These peptide motifs mimicking not only linear but conformational and carbohydrate epitopes of viruses and bacteria can be used as biomarkers for diagnosis of infectious diseases. The costs of analyzing large number of serum samples using high throughput sequencing can be essentially decreased by multiplexing the samples using PCR primers containing DNA indexes tagged to serum samples.

## Materials and Methods

### Ethics Statement

The archived de-identified human serum samples for this study were accrued from the RPCI Data Bank and BioRepository (DDBR) with the approval of the Office of Research Subject Protection protocol #NHR010810. Written informed consent from all the donors to DDBR was obtained for use the clinical samples in research. Human serum samples were accrued with the approval of the Institutional Review Board committee of the Roswell Park Cancer Institute. The work with mice was approved by the IACUC committee of the University of Maryland School of Medicine.

### Immunizations and ELISA

Mice were immunized subcutaneously with proteins in complete Freund’s adjuvant as described previously [10], [11]. The mouse sera at 500-fold dilution were incubated with the overlapping peptides covalently immobilized to the amine-binding, maleic-anhydride activated plates (Pierce) and the antibody binding to peptides were measured using secondary antibodies labeled with horse radish peroxidase.

### Western Blot

Whole cell lysate of 293T cells over-expressing recombinant CPA3 protein or the control lysate of the 293T cells transfected with empty vector were separated on the 10% SDS-PAGE acrylamide gel electrophoresis and transferred to Immobilon PVDF membrane (Millipore). After blocking the membrane with 5% milk in phosphate buffered saline buffer (PBS) the membrane was incubated overnight at 4°C with serum from either melanoma patient or healthy donor. Serum was diluted 100-fold in PBS containing 0.05% Tween 20 and 1% bovine serum albumin. A horseradish peroxidase-conjugated goat anti-human antibody was then added, and secondary antibodies were detected through autoradiography using enhanced chemiluminescence (ECL Plus, General Electric Healthcare, Milwaukee, WI).

### Generating Serum Antibody Repertoire Profiles

Twenty µl of mouse or human serum and 10 µl of the Ph.D.7 random peptide library (NEB) were diluted in 200 µl of the Tris Buffered Saline (TBST) buffer containing 0.1% Tween 20 and 1% BSA and incubated overnight at room temperature. The phages bound to antibodies were isolated as follows. 20 µl of protein G agarose beads (Santa Cruz) were added to the phage –antibody mixture and incubated for 1 hr. To eliminate the unbound phage the mixture with beads was transferred to the well of 96-well MultiScreen-Mesh Filter plate (Millipore) containing 20 µm pore size nylon mesh at the bottom. The unbound phage was removed by applying vacuum to the outside of the nylon mesh using micropipette tip. The beads were washed 4 times by adding to the well 100 µl of TBST buffer and removing the liquid by applying vacuum to the outside of the nylon mesh using micropipette tip. The phage bound to the antibodies was eluted by adding to the beads of 100 µl of 100 mM Tris-glycine buffer pH 2.2 followed by neutralization using 20 µl 1 M Tris buffer pH 9.1. The eluted phages were used for the amplification in bacteria. The amplified phages were incubated with 20 µl of serum overnight at room temperature followed by isolation of the antibodies-bound phages using 20 µl of protein G agarose beads. The phages were then eluted from antibodies/protein G complexes using low pH buffer as described above, and the DNA was isolated using phenol-chloroform extraction and ethanol precipitation. The 21 nt long DNA fragments coding for random peptides were PCR-amplified using primers containing a sequence for annealing to the Illumina flow cell and the sequence complementary to the Illumina sequencing primer. The PCR-amplified DNA library was purified on agarose gel and DNA from all samples were multiplexed by adding 4-base bar code at the beginning of each DNA fragment.

### Nextgen Data Processing

The high-throughput sequencing was performed using Illumina Hiseq2000. A total of about 313 million raw reads were generated. The sequences were de-multiplexed to determine its source sample. The 21- base nucleotides representing the 7-amino-acid peptide were extracted between base position 29 and 49. All identical 21-mer DNA sequences were collapsed into single sequence with its coverage (frequency) recorded in the result multi-FASTA file. These DNA sequences were translated to peptide sequences using the first frame. All barcode splitting, read trimming, and sequence collapsing were done using FASTX-Toolkit. Peptide translation, selection of subsets of sequences of shared DNA, abundant DNA passing minimum coverage threshold, were done through customized Perl script. Bl2seq was also automated to handle batch processing of thousands of sequences in a very short time frame. The next generation sequencing data are deposited to the NIH Short Read Archive. The accession number for the sequences in the SRA database is SRP021104.

## Supporting Information

Table S1 The table shows 500 the most abundant peptides with corresponding copy numbers selected for the antibodies from each of the four anti-PAP sera. The selected peptides are not shared by the sera from unimmunized mice as well as from mice immunized with the PSA antigen.
(DOC)

Tables S2 S2A, S2B and S2C. The table shows the list of proteins selected by doing protein BLAST of peptide sequences against refseq_protein database for the Homo Sapiens (taxid:9606) with the maximal score ≥18.5 threshold parameter. In the column A, the proteins are sorted by the increase of the protein accession number. Highlighted in yellow are the proteins which have been retrieved multiple number of times by BLAST search against different peptides. The column B shows the lengths of proteins in the number of amino acids. The column C shows the number of the matches to peptides that retrieved proteins at the selected E-value threshold. The column D shows the initial scores calculated as the number of matches in column C divided by protein length in column B. Sorting the data by the descending numbers in column D allowed to select the top candidates for the second step of analysis. The column E shows the sums of scores for all the peptides that produced matches to the protein in Blast2seq analysis of the protein against all the most abundant 500 peptides. The column F shows the final scores calculated as the sums overall scores in column E divided by protein length in column B. Sorting the data by the descending numbers in column E allows to select the candidate antigens containing linear epitopes recognized by serum antibodies. The column G shows the sum of the scores for peptides that match to the single major site on the protein.
(XLS)
