# Supplementary material for: Serum Antibody Repertoire Profiling Using In Silico Antigen Screen
Source: PLoS One. 2013 Jun 27;8(6):e67181. doi: 10.1371/journal.pone.0067181 (PMC3695087; doi:10.1371/journal.pone.0067181)
Supplement: Table S1 — The table shows 500 the most abundant peptides with corresponding copy numbers selected for the antibodies from each of the four anti-PAP sera. The selected peptides are not shared by the sera from unimmunized mice as well as from mice immunized with the PSA antigen. (DOC) [file pone.0067181.s001.doc]

**Supplementary Table S1.**

| Antiserum | PAP1 | PAP2 | PAP3 | PAP4 |
| --- | --- | --- | --- | --- |
| Peptide sequences with corresponding copy numbers | >213-8341_1 IVLPTWL >236-7646_1 YSTRHTD >280-6564_1 ADRSPLW >319-5936_1 YSSVHID >355-5487_1 YTSQTPR >406-4879_1 QTSITMY >426-4754_1 HRVLWHQ >436-4720_1 SFTYPSW >439-4684_1 ELCFKCS >501-4296_1 SDRSALW >502-4286_1 VSPGPTG >508-4251_1 SEWLWDH >515-4208_1 QCTLCPR >520-4188_1 YATPWQM >545-3992_1 TLIDPSR >585-3803_1 VLSDPPP >595-3753_1 RIPMQDL >611-3641_1 YVFPTWS >619-3619_1 ERMNHNP >643-3466_1 FYLPPWS >646-3434_1 ASQDITP >649-3422_1 IHTHPWY >679-3345_1 SHGSVHA >705-3237_1 GLGSVKP >714-3209_1 YIWPTWA >716-3205_1 VGFRLGL >731-3137_1 STTIALF >745-3081_1 SSPPKRL >746-3080_1 YAPPYQR >749-3060_1 STSELYG >772-2990_1 YDMSLIG >818-2853_1 QYMNQHA >835-2808_1 YDRSVLW >839-2802_1 HFVWTRS >847-2777_1 IELRGIY >849-2777_1 SDTTGLP >853-2770_1 EFTYPSW >875-2721_1 GWMMRFS >892-2684_1 QDRSILW >894-2683_1 ERVMSMM >920-2624_1 LAAKVHR >936-2593_1 MLDPRWE >944-2574_1 NFMYPSW >966-2524_1 TPMKISH >969-2519_1 AQSNSMH >971-2514_1 ILPVHVS >983-2495_1 VPPGTII >986-2487_1 QGLGTRG >991-2474_1 EPKTKHI >1016-2403_1 HLSYGLN >1017-2399_1 YIFPTWA >1047-2361_1 YVFPTWM >1069-2333_1 QFMSQVY >1074-2324_1 SSGQHAF >1084-2307_1 FIWPTWS >1120-2238_1 TGSPQAR >1124-2235_1 YGVMAPH >1125-2234_1 RNDLPYT >1127-2231_1 GQWSRIN >1155-2183_1 GNNLVRT >1156-2182_1 SSAQSSQ >1168-2165_1 FKFTFAL >1178-2152_1 SRYVDGY >1187-2142_1 VPSRESW >1211-2092_1 QLAMTYV >1231-2061_1 MMNPIRN >1248-2041_1 GELPGTA >1249-2037_1 WIGIDTV >1253-2030_1 LSINVVF >1265-2018_1 EEWNWLR >1283-1998_1 LSWTGKS >1317-1959_1 GLFREGW >1335-1930_1 MAQSSAA >1348-1918_1 QYMYQLP >1350-1916_1 DFWHHLR >1357-1900_1 ADRSALF >1369-1885_1 VPSFVPL >1415-1820_1 QCLLTCA >1424-1813_1 DSPLHAT >1439-1803_1 QTYRPSY >1444-1800_1 ANFWGES >1457-1784_1 NFYLPTW >1496-1734_1 WPISFLI >1497-1734_1 YVFPTWM >1500-1731_1 YSTNHID >1504-1724_1 WKSPVLM >1513-1716_1 VFGVHAH >1520-1711_1 FNQPTWL >1522-1710_1 HHSPMIG >1527-1706_1 SLGKTVA >1537-1691_1 AWPRHAA >1540-1686_1 GIFKYFS >1542-1684_1 NFVPPSW >1555-1663_1 MGDIKHV >1561-1658_1 TGTDATF >1562-1655_1 CYMCQSL >1577-1641_1 QYMFQTS >1585-1636_1 TQGYATS >1606-1622_1 NSGLHMF >1619-1603_1 YPHRQVP >1635-1589_1 YSRLHLD >1637-1586_1 LPVERLN >1648-1578_1 LLAVDAL >1649-1578_1 HSPQSIA >1652-1576_1 SGMTVLI >1657-1573_1 WDPHWPK >1665-1565_1 VVTIPYP >1667-1563_1 LATRSSS >1675-1556_1 GPHFELR >1683-1551_1 RVLPSDE >1689-1544_1 ETSSSIA >1691-1544_1 HLQSSYQ >1701-1528_1 SIRLHLG >1702-1526_1 NGYINAM >1720-1506_1 FYLPTWY >1735-1494_1 EFHLGES >1739-1492_1 MSNRDSE >1740-1491_1 FTLPSWL >1745-1489_1 KVTTMKP >1746-1488_1 APPLSNL  >1762-1481_1 GSAQESH >1763-1480_1 HHGAKHF >1800-1452_1 TEFDVDH >1824-1436_1 ITSPWPG >1852-1413_1 VAKTYTS >1864-1407_1 WGQNQSS >1866-1406_1 LLGNIPP >1869-1404_1 TSTTSRG >1885-1391_1 YPFGVWT >1889-1389_1 NHHAYPT >1899-1380_1 ELALWST >1900-1380_1 SWLSIFG >1914-1369_1 TDNSARP >1924-1363_1 HTAHVVT >1927-1361_1 TVCCQTA >1946-1351_1 FWFPPWE >1948-1350_1 KFIIKAQ >1952-1347_1 SVELQFG >1981-1335_1 FYWPTWV >1989-1330_1 MTQDITI >1990-1330_1 SPLPWWQ >2024-1305_1 WGGTLAR >2025-1304_1 FSTVSQR >2030-1301_1 RLVAQWP >2034-1298_1 WTPMHDP >2041-1296_1 GVQKASN >2042-1295_1 DRLHMEP >2048-1290_1 QFFWPAG >2057-1285_1 WSSGVIF >2058-1284_1 SLALMMR >2063-1280_1 ETHHAHR >2069-1277_1 LSHFSIV >2078-1274_1 FIPRLSY >2098-1261_1 SVTQSVI >2116-1250_1 LMTPIMK >2129-1242_1 KFVVKFN >2156-1226_1 DHDRSWF >2161-1224_1 TQHSYFL >2164-1222_1 GNTYGFH >2168-1217_1 IVMPPGP >2170-1216_1 VSQSHLQ >2180-1210_1 FRIPMID >2183-1208_1 IFARTTM >2184-1208_1 QHRALPT >2190-1206_1 AVNTFML >2191-1206_1 KGAYYQS >2205-1197_1 VGLSIHI >2229-1185_1 DKLYVTL >2231-1183_1 TVRTGST >2253-1169_1 GQDITAQ >2256-1169_1 ADRSIMW >2273-1155_1 THHHPDE >2276-1151_1 QQFKSMS >2278-1148_1 LIPTNTV >2281-1147_1 LGTILHQ >2286-1144_1 VHTGGLA >2288-1143_1 IKISYPS >2291-1143_1 MPGRGGV >2292-1143_1 ATFGVWT >2302-1137_1 NHVQGLP >2304-1134_1 QFMSQMA >2309-1131_1 IKLQPSV >2321-1124_1 ETTQRPM >2328-1117_1 GPYHAQP >2346-1106_1 FTMPPWE >2352-1103_1 FPFKWLA >2354-1102_1 GASRAPA >2384-1088_1 ADHVSHG >2387-1085_1 VTSNFSH >2388-1085_1 NSLTSYT >2389-1085_1 MLERFLS >2397-1080_1 HYFRING >2401-1078_1 TFTYPTW >2406-1074_1 HTHWFHT >2414-1070_1 DPKGAYT >2433-1061_1 YSSMHID >2453-1052_1 IGQLVKL >2460-1050_1 DSEHMTS >2463-1048_1 WTWPSWA >2465-1047_1 YTSQTIR >2473-1042_1 KFWQGVT >2477-1041_1 SKQDIIA >2490-1031_1 SGMDRHE >2491-1031_1 VTANTRE >2503-1025_1 FSFTLTV >2505-1024_1 AKVAITI >2509-1022_1 FVRPTWH >2511-1021_1 VSGTHML >2512-1021_1 SPFFWEL >2517-1019_1 TARSVWI >2518-1019_1 VSTRHLN >2534-1013_1 WNAPVPN >2547-1006_1 NLDANLV >2548-1006_1 KPNFDLH >2551-1004_1 GAAGDAY >2557-1002_1 TMTRNFM >2575-995_1 LRLNITW >2581-992_1 RIPMNDT >2582-991_1 YTGLQTQ >2595-985_1 CIFTCHD >2596-985_1 MDRSALW >2608-980_1 TISKWEE >2618-973_1 DRYVSWH >2620-973_1 EMRTERV >2624-970_1 DWNKAGD >2625-970_1 CHLYCEA >2630-969_1 GEWEYDH >2633-966_1 WNGMIGM >2634-966_1 EQTQARV >2638-963_1 YSTTHVD >2644-962_1 MPAHLNE >2646-962_1 SPFKWLA >2656-958_1 HISWRMM >2661-957_1 QEYHLAS >2667-955_1 LLGGAVA >2670-954_1 RIPMTDE >2675-953_1 NICIRCP >2677-952_1 QNWVPFN >2680-951_1 CYMCYQT >2682-951_1 SVVLNWN >2684-950_1 QFMYQPV >2687-948_1 YTTLHRD >2688-947_1 LANMSLL >2703-942_1 FVEPRSH >2704-942_1 SSGELWN >2712-939_1 MTDSHVS >2716-937_1 SYQMDYA >2733-932_1 AAKLLPH >2738-931_1 GRIGLQV >2741-929_1 QYMN*NF >2742-929_1 MPALVKF >2751-925_1 NLTHRGP >2757-922_1 LSLNMHD >2759-921_1 VLDFTLA >2762-920_1 TSYVGIY >2769-917_1 YSVLHLD >2778-914_1 YTSNHLD >2781-912_1 KFVVKHT >2785-911_1 HSREFTF >2788-908_1 SHRYDPG >2792-907_1 DDRLNAL >2796-905_1 RCESMQC >2814-899_1 LHLRLSA >2819-897_1 SKPNTVS >2823-896_1 AHLAADM >2824-896_1 GASHHSK >2829-895_1 *TPTQQY >2831-894_1 ESQSLCV >2832-894_1 TNPLELP >2835-892_1 KPQLNYF >2838-891_1 GLLTYGT >2849-887_1 SHTNPKS >2851-887_1 HLKAQFN >2879-876_1 FGSRVPP >2887-872_1 QNNPYLH >2896-867_1 VYNSSHM >2898-866_1 TVVMYPG >2900-866_1 FWYPTFL >2902-865_1 WNWPTWE >2905-865_1 FSLAGGR >2914-862_1 IPTWMTK >2919-861_1 QFMAQKY >2931-856_1 HPATICR >2934-856_1 MLVEATD >2936-855_1 RTPSELK >2937-855_1 HLLSLAR >2941-854_1 NGTVGYL >2946-852_1 LKATVSI >2950-850_1 GGIKVTL >2952-850_1 HKLHLNI >2957-849_1 TVFPWMI >2959-847_1 NDPAGHF >2967-845_1 AVHLPYR >2994-834_1 SIVMART >3000-831_1 VGWIGKL >3001-831_1 QFMAQVS >3015-827_1 YESTPEW >3031-822_1 VLLPTRI >3033-820_1 QYFASSD >3054-813_1 RPITFHL >3057-813_1 YTCSLCG >3065-810_1 HAMATTV >3068-809_1 GWWPLLK >3078-806_1 VDGYTNW >3085-804_1 TDSMKNM >3088-804_1 WPIPPHS >3102-800_1 VSAAFVS >3108-799_1 NVFITHR >3109-798_1 FTGRMAI >3119-795_1 LVRVTVN >3120-794_1 TVLRHDT >3123-793_1 TTVMAWN >3124-792_1 CYMCMTF >3137-789_1 QYMF*MT >3143-786_1 LCYLFCE >3156-782_1 SHTIVQH >3157-782_1 TADPTIR >3161-782_1 YEATLVL >3163-781_1 WDAWWRH >3165-781_1 WSTTHYN >3168-780_1 HFRTMSA >3169-780_1 HSSFHDM >3186-776_1 WWVPPTW >3196-773_1 VRATISI >3199-773_1 NKTMLEL >3201-772_1 VI*TITR >3204-772_1 LKGSSPL >3209-771_1 AFVQPSW >3212-771_1 SFGPCCS >3218-770_1 QFMHQTL >3220-769_1 SHDNDRQ >3225-768_1 ARDSSFV >3233-765_1 VGPGIGK >3236-764_1 FVLPSWI >3240-763_1 DAVQPPI >3242-762_1 MAAKLVT >3245-761_1 TTSWPPL >3253-759_1 QPYNMNW >3254-759_1 TIPKEHY >3256-758_1 NVWNYSR >3260-756_1 LAARIPK >3266-755_1 GYSNTGK >3292-748_1 TFVMPSC >3296-746_1 ATESTQL >3310-743_1 YVLPSWM >3312-741_1 MASYEMS >3313-741_1 MLDILHL >3317-738_1 GAQVLYP >3326-734_1 YETSMHQ >3327-734_1 SAYVADK >3343-730_1 VIPPWWS >3355-727_1 AHHLAKD >3358-726_1 FVIPSWA >3365-724_1 LYRPDYD >3371-722_1 TLFSLNV >3375-720_1 FTSRVHV >3380-718_1 HAAHQPP >3384-718_1 TLRAPET >3385-717_1 AFQQRAP >3389-716_1 VYNHVGT >3394-715_1 NEPARHS >3397-714_1 DKMPRDS >3399-713_1 HSSFITV >3404-712_1 IQWPTWL >3405-712_1 FSLPTWL >3406-711_1 WHTNNTV >3410-710_1 DVPNWRT >3415-708_1 SKFNLAY >3416-708_1 AQTTRHH >3420-708_1 SGLDLWT >3429-706_1 TPDGSGL >3432-706_1 QVSMLST  >3438-704_1 KLISSWS  >3442-703_1 ITKSMSG >3451-701_1 QFMFQDN >3456-701_1 GDRSVLW >3459-700_1 LPLTYDA >3460-700_1 TFASVNA >3464-699_1 LVARVAG >3473-696_1 TSLRIHL >3475-696_1 VTSMSDN >3477-696_1 MTVAVTS >3484-695_1 MSSSLKS >3491-693_1 SLH*TIR >3500-692_1 WTSYLGG >3505-690_1 WTTLAFY >3509-689_1 HPKAPEP >3514-689_1 QYMFQWA >3523-687_1 GGGLLPS >3524-687_1 HLTLVAS >3525-687_1 LTMTYDS >3531-684_1 LTYRAAA >3536-683_1 GSFGIYT >3538-683_1 LIATPHR >3540-682_1 AEHYCCV >3543-681_1 TYFAEHK >3551-679_1 SWPWYYQ >3557-678_1 LAPRAVS >3560-678_1 >3565-676_1 GSAVPWT >3568-676_1 HTSLTMR >3569-675_1 FPRYHYL >3576-674_1 KSSYDFM >3578-673_1 VAHPSMP >3588-671_1 QAAASHS >3589-670_1 WPGLIPV >3595-669_1 NYMLPWA >3601-666_1 YWYQHRF >3609-664_1 WTAGLQI >3611-664_1 YWSEHRA >3614-662_1 SWSDFLK >3619-661_1 VAPDHKR >3620-660_1 QEMGSAR >3631-657_1 SGRADET >3646-654_1 HAKVGIY >3647-653_1 WNIEHLL >3680-648_1 GTFGIRT >3681-648_1 YTHNHHD >3682-647_1 YTTQHLD >3689-645_1 VPDPTQQ >3693-644_1 HMVTAGK >3696-644_1 QPVEIRR >3707-641_1 YTSSHVD >3713-639_1 SSSEYTS >3716-639_1 VPATVKY >3725-637_1 GSAQPSR >3729-637_1 NLFAIYM >3730-637_1 ATDSFAT >3736-636_1 GTHPREW >3756-632_1 KFFVKYT >3757-632_1 AWDSRML >3767-630_1 TLGHSRQ >3769-630_1 QMNENTY >3776-628_1 DFIPHTL >3785-626_1 FVHPSWS >3795-624_1 DKHRPFW >3800-622_1 YSTLH*D >3808-619_1 NMPAGVR >3809-619_1 AGFRLVL >3810-619_1 YSWKHYD >3815-618_1 ASTEWLA >3818-617_1 NTRIIVS >3832-614_1 GWHHKLI >3835-613_1 ADHLNVL >3837-613_1 GWMTTFS >3844-611_1 YSYKGYA >3848-610_1 QFMYQPS >3850-610_1 GWMVTFM >3851-610_1 DRAPHQL >3854-609_1 LQLQVLV >3856-609_1 SIWHLGA 130-132 >3859-608_1 TQVFMVE >3860-608_1 EQTWAMR >3864-608_1 YPFKWLN >3872-606_1 HLLNGVN >3874-606_1 CYVWRCD >3883-604_1 EGFWLAI >3884-604_1 AAHQKEQ >3889-603_1 MSVSLNA >3892-602_1 HIITLKH >3897-601_1 IRQPQFN >3898-600_1 YTSQTWR >3915-596_1 NVPVGTF >3918-595_1 CFMCYSD >3921-595_1 SGAERFQ >3922-595_1 NVVTTLR >3924-594_1 VNFQIRV >3927-594_1 STWELPR >3932-593_1 FKQHATA >3934-593_1 ASVHSVH >3935-593_1 ACHTRHC >3936-592_1 TNTTGWR >3947-589_1 TMARIPR >3950-589_1 CYFKWQC >3951-589_1 QSGILYT >3954-588_1 TQLNIWL >3955-588_1 SPWPLMT >3974-585_1 SEPSMQQ >3983-584_1 VSYAHEM >3991-582_1 GTRPNTV >3993-582_1 FTAAIYS >3996-581_1 QALWFRV >3998-581_1 WNSLVRS >4004-579_1 DSFWRWP >4005-579_1 RTDLARH >4012-578_1 FANPSLM >4021-576_1 ARFVTNA >4024-575_1 MSQGNQA >4030-575_1 VFVLPSW >4031-574_1 EPQHQPM >4036-574_1 GLFTVTV >4039-573_1 GPLAFRW >4043-573_1 MLHSTVS >4052-572_1 CYMCRML >4058-571_1 WPDLRTY >4066-570_1 DGTYTHV >4079-567_1 DHRTVDN >4082-566_1 LLAKRIV >4087-565_1 KFIVKSM >4089-565_1 LAMQLSD | >133-13001_1 AWFYPPW >156-11910_1 YVHPPWW >157-11908_1 LGYPWMQ >166-11423_1 NFRLPPW >178-10841_1 NPQWQTS >217-9446_1 YELPPWM >225-9164_1 FVRPSWI >229-8978_1 AFFGPSW >255-8115_1 WTRPPWE >263-8001_1 TFLPSWA >264-7974_1 YVFPTWS >267-7948_1 APPLFTP >302-7088_1 WTLPSWE >303-7032_1 NFQMPSW >318-6902_1 QWWNHEI >323-6818_1 GCCMHKN >355-6308_1 YINPSWW >363-6226_1 SLVDIWV >364-6210_1 FSLPPWA >371-6099_1 YVWPSWM >376-6041_1 GSLHFLV >380-6015_1 FTSPSWQ >389-5925_1 TYWPFPR >393-5893_1 SPLNIAR >397-5841_1 SYPWLIN >419-5674_1 KDLRDWF >437-5566_1 YVNPWEP >454-5382_1 FWEPPWG >455-5372_1 GDMHRVM >456-5363_1 TQVRPPW >465-5268_1 SFLPPWM >473-5176_1 WNWPTWE >488-5067_1 DLYYGRV >507-4925_1 GLWADNE >509-4911_1 FNSPSWS >516-4858_1 GFMAPSW >518-4846_1 LGSAWDV >525-4793_1 IIMPSWE >526-4787_1 GLNAALF >535-4734_1 APQMHLP >536-4722_1 TDGKSHL >551-4575_1 YVQPSWL >557-4543_1 WEKINHT >561-4519_1 AP*MHLP >569-4470_1 YLLPPWE >572-4457_1 HYLPSWH >584-4396_1 FSFPPWS >586-4391_1 LVLPSWL >611-4236_1 EQFYPAM >625-4170_1 ILWLLLW >626-4159_1 NYWPFPW >627-4151_1 VVWDRNP >629-4140_1 GYPWISN >640-4083_1 ESLNSFA >645-4062_1 TMWPFPS >649-4044_1 FVRPTWH >650-4042_1 HPLQVFV >653-4026_1 YFLPPWI >656-4007_1 NVLPPWL >662-3993_1 FELPSWM >664-3987_1 FVWPYWQ >675-3935_1 IVLPSWW >676-3935_1 APPMHII >683-3923_1 EFFFPSF >686-3907_1 VYAHTWL >687-3906_1 FVSPSWK >694-3871_1 FSMPSWQ >696-3868_1 ITLPTWL >699-3863_1 GFKEPSW >700-3861_1 TFRAPSW >711-3797_1 KAPHLES >722-3764_1 HFAFPSW >730-3721_1 FHRPSWD >732-3710_1 FTHPSWS >742-3676_1 VFVEPSW >743-3661_1 GGKLPLN >744-3657_1 SQLMPSW >749-3641_1 IWLPPWF >752-3628_1 FLLPPWN >766-3561_1 GWFLPSW >768-3551_1 WFINPTW >771-3539_1 AWWAPSW >779-3509_1 SFVKPTW >784-3499_1 KPVDLTA >785-3490_1 FRQPSWY >787-3481_1 AQWYPPA >804-3422_1 WVLPSWM >807-3405_1 FFDPSWN >809-3399_1 GFTLPSW >816-3384_1 FDFPPWA >818-3373_1 FNEPPWH >831-3340_1 VWMPSWA >835-3329_1 HEPTVMS >842-3285_1 RMVLPSW >858-3222_1 LTDHQRY >863-3215_1 NFILPTW >865-3208_1 ANLSQVL >872-3191_1 GFFLPTW >882-3172_1 SDHRHWI >884-3165_1 RFMVPSW >899-3114_1 VQMPASK >910-3080_1 HSLERLA >922-3036_1 FALPSWH >925-3029_1 GDYIPVK >927-3028_1 EDTKTSH >939-2996_1 FVFPSFH >940-2995_1 WTQQWYY >941-2994_1 LVYGDER >949-2972_1 VPSGKLH >955-2945_1 VVWDRHL >959-2935_1 ILLPSWE >963-2920_1 TFSYPTW >966-2909_1 VHNTPYQ >967-2902_1 QWWNLEI >976-2877_1 WHDPSWV >981-2861_1 DFIIPSW >983-2853_1 YPWINQP >1006-2796_1 RFMDPPW >1023-2760_1 FNPPSWG >1026-2758_1 FINPTWA >1027-2754_1 TWFAPTW >1030-2748_1 VILPSWY >1034-2744_1 VPGHHLM >1041-2737_1 SVPSSEV >1043-2732_1 FVMPYWA >1048-2728_1 FKWPPWN >1052-2722_1 LIYPSWA >1058-2713_1 KSADHYY >1061-2705_1 FSLQSFA >1070-2682_1 SFMFPPW >1071-2679_1 SFYEPSW >1072-2671_1 TFTLPTW >1073-2669_1 HSLEMLW >1080-2660_1 SFQSPSW >1081-2660_1 SYLRDLW >1084-2652_1 HTHASGG >1085-2650_1 AFRMPSW >1092-2643_1 LVWDPWP >1101-2625_1 FTQPSWQ >1119-2575_1 RWVSPPW >1125-2561_1 VLPSWAQ >1132-2551_1 LIPVALT >1140-2544_1 YWLPPWS >1147-2538_1 EQFYPNW >1150-2532_1 HVKAVET >1160-2512_1 YEMPIPL >1170-2497_1 GASRAPA >1176-2480_1 NFTRPSW >1181-2472_1 VVWDRSP >1187-2461_1 MVVSSAN >1190-2458_1 FTQPPWR >1191-2458_1 FLVPSWH >1193-2455_1 WFRMPSW >1195-2451_1 QMEALMP >1197-2450_1 SPPLQMF >1200-2439_1 EFRSPSW >1203-2434_1 IFSFPSW >1206-2426_1 EVFWARL >1207-2423_1 RPVDTPQ >1214-2408_1 HSLVLPN >1220-2402_1 QWWAGES >1241-2378_1 KFQLPSW >1243-2376_1 TFNPPPW >1247-2373_1 YPWMQFS >1252-2362_1 FLWPSWT >1254-2357_1 QFRLPPW >1267-2331_1 FNPMWQW >1274-2323_1 FHYPSWR >1275-2322_1 FTEPTWA >1279-2317_1 VPRWFPP >1281-2316_1 YYFPSWM >1282-2315_1 YIVLPPW >1283-2314_1 FFSPSWL >1286-2311_1 RADYHAY >1292-2289_1 YLGNYMI >1302-2281_1 HLSTKGV >1314-2258_1 TWWMREL >1316-2254_1 FYSPSWT >1323-2246_1 WPIFWH* >1343-2220_1 QLTLPSW >1356-2206_1 YSGQFNW >1360-2203_1 NFAFPSW >1361-2202_1 HFNEPSW >1362-2200_1 ISTHYYL >1364-2197_1 IPLNYNV >1367-2190_1 FISPSWT >1370-2186_1 NNNPQWQ >1372-2183_1 FVAPSWN >1378-2172_1 LVWPTWA >1384-2166_1 WSWPSWV >1385-2165_1 SLIQPSW >1388-2162_1 QVLPTWS >1389-2161_1 TYVYPPW >1390-2160_1 TNPMW*M >1391-2160_1 HSLEDVY >1397-2147_1 LVNPPWA >1398-2147_1 HMMRTNH >1399-2147_1 LDFPFTS >1402-2143_1 HVLPPWS >1404-2141_1 WSEFRTL >1408-2137_1 HSLEAIH >1409-2136_1 SHYQYPD >1413-2130_1 HKGFPMS >1417-2124_1 LYLPPWQ >1418-2123_1 LNLPTWM >1419-2122_1 FLNPSWQ >1421-2118_1 WFMPPWD >1426-2107_1 DYLQNNA >1427-2107_1 HSKPYLT >1429-2102_1 FIRPTWG >1431-2098_1 YHWATWE >1435-2096_1 HFTMPSW >1447-2086_1 YPEWGHN >1448-2086_1 DQPWHWI >1450-2080_1 YDMSLIG >1455-2073_1 EFISPPW >1479-2050_1 QVLPSWA >1487-2036_1 HALEMIA >1488-2036_1 WSTYRTA >1492-2032_1 VTNFKTN >1499-2016_1 LILPPWL >1503-2007_1 WSWPSWN >1504-2007_1 TRLYITD >1507-2004_1 HIGQPRS >1512-1997_1 VEDYMMR >1518-1989_1 WQRLPSW >1521-1984_1 YVRPPWS >1523-1983_1 MVNPTWM >1527-1973_1 LLWDPFP >1528-1973_1 NVSLQMF >1529-1972_1 QWHEPSW >1530-1970_1 LWWSPSW >1538-1955_1 NDAFASM >1547-1951_1 YIMPSWS >1549-1949_1 FEWPSWG >1556-1936_1 FMNPSWK >1562-1930_1 FIKPSWV >1563-1929_1 HHIDSLW >1570-1922_1 WSLHRTG >1579-1912_1 YNWVQQS >1580-1912_1 FRMPSWT >1586-1908_1 NTVVLHL >1590-1905_1 YVLPSWM >1599-1895_1 HSLTIRN >1603-1892_1 TYPWVTQ >1620-1878_1 NWLPTWE >1623-1877_1 FLNPSWE >1627-1875_1 NWMWDAN >1635-1862_1 AFIGPPW >1637-1860_1 EIAMTTL >1640-1855_1 LHHGVPV >1644-1852_1 INLPSWM >1650-1840_1 WTYPPWG >1657-1833_1 HFTHPSC >1661-1828_1 IIGPGWF >1662-1825_1 WHTSIPL >1664-1820_1 TFTNPPW >1665-1819_1 GHYLPLS >1666-1819_1 AQWYPFS >1670-1814_1 FYLPTWY >1675-1809_1 AWDLFRA >1676-1808_1 QWWNREL >1678-1808_1 QTGDSTH >1681-1807_1 LHKGLAV >1684-1804_1 VFQRPSW >1686-1803_1 SWNPKER >1692-1798_1 FTPLWHL >1698-1792_1 VFWDSLG >1705-1779_1 SFWPFPS >1706-1779_1 WIIPPWL >1710-1773_1 IVAHVVA >1712-1770_1 SALPKSL >1713-1770_1 WVAPSWQ >1729-1752_1 EYWPVPF >1738-1746_1 TTPTNTY >1740-1745_1 SYPWMEQ >1741-1743_1 IRMPSWA >1747-1738_1 FNTPPWE >1748-1737_1 FTVPPWD >1753-1733_1 TMQTFSR >1754-1731_1 SWTWPSW >1755-1731_1 AWVSPPW >1758-1728_1 TDPLSIT >1767-1718_1 HLSVGSL >1771-1715_1 FMLPTWN >1778-1708_1 HSLELIH >1781-1704_1 DRWNTNV >1783-1703_1 QFLPPPW >1794-1690_1 TADAGVT >1795-1689_1 FFVGPPW >1797-1686_1 LSNPQWQ >1805-1677_1 FQLPSWN >1815-1666_1 NVLPPWA >1818-1665_1 NPSLQTF >1824-1660_1 DLHVFSN >1827-1658_1 FNQPTWL >1829-1655_1 TVSHANG >1838-1644_1 AFRAPTW >1841-1642_1 HSLEWLS >1842-1641_1 DYSLPWW >1855-1627_1 SFSYPTW >1857-1623_1 APVIGLY >1867-1617_1 LLREPNY >1877-1605_1 FTTPSWH >1882-1600_1 QPFESWS >1894-1587_1 HALEFIS >1899-1584_1 HFLQPSW >1902-1579_1 SQVEPPW >1916-1565_1 VDSVHVR >1919-1565_1 QMNENTY >1924-1559_1 QPNVNNY >1927-1555_1 WSMFRTL >1932-1550_1 HHVQNSY >1933-1549_1 GGIMLWW >1941-1543_1 FQVGPLT >1948-1539_1 FTSPTWM >1949-1539_1 KPLMQSL >1952-1538_1 SFTQPTW >1970-1520_1 LFLPPWS >1973-1519_1 FPPILHI >1974-1518_1 NQFMPSW >1992-1500_1 SFKFPTW >1993-1499_1 NNGPISK >2013-1482_1 DTPMWHL >2014-1482_1 LPWTDSS >2018-1480_1 RFIMPSW >2021-1478_1 LTKISTP >2022-1477_1 YIIPPWL >2023-1476_1 YVMPSWT >2024-1476_1 QQLLPPW >2042-1466_1 QFTAPSW >2043-1464_1 LEAHILL >2045-1456_1 FMRPSWM >2051-1450_1 LYIEPSW >2057-1447_1 LWWQPYP >2060-1443_1 KTSVDYD >2062-1441_1 TNDRYMI >2063-1441_1 HHTSRPI >2065-1439_1 NEQMPLY >2068-1438_1 YYMPSWS >2069-1437_1 LELPPWL >2070-1437_1 EYVWSRS >2082-1430_1 DLHNWGQ >2083-1429_1 VLASVYR >2084-1429_1 YVFPSWY >2085-1429_1 GPEHTNP >2100-1418_1 EWMPSWA >2111-1412_1 FIEPSWW >2116-1409_1 FQLPPWH >2121-1407_1 KFTIPSW >2123-1407_1 SQISPSW >2125-1405_1 IFLPSWL >2131-1403_1 FVYPTWT >2134-1403_1 LESASWR >2135-1402_1 NLSSVHY >2140-1399_1 RLVLPPW >2141-1399_1 EPARH*N >2144-1397_1 NRLWDSF >2150-1390_1 TYSLPWW >2156-1387_1 EHLGSYF >2159-1385_1 WSEFRTK >2161-1384_1 LFSEPSW >2168-1379_1 HNVSPPW >2169-1379_1 HSLQFIG >2170-1379_1 HSLQLIF >2179-1375_1 TSTFSDK >2181-1374_1 QHVMPSW >2182-1373_1 NFLRPSW >2188-1370_1 YAPPYQR >2189-1368_1 SYTVHIL >2190-1366_1 YIFPTWA >2194-1362_1 SFTYPSW >2199-1360_1 KFISPPW >2200-1359_1 GQFMPPW >2203-1358_1 IYYPSWV >2204-1357_1 LTLPPWL >2206-1356_1 NLIPPPW >2214-1349_1 RQLDVIA >2216-1347_1 GLMLPPW >2219-1345_1 TGTYGWT >2222-1343_1 NPMWQGR >2224-1340_1 SFWPFPK >2232-1334_1 YNFPPWA >2237-1330_1 QPDSNVA >2242-1327_1 HGMFLLD >2244-1326_1 VLIPPPW >2246-1325_1 YVLPPWL >2248-1323_1 DQFYPLH >2249-1322_1 NFIDPPW >2250-1321_1 QFLPPWS >2255-1321_1 QTVKNTF >2261-1318_1 IVHPTWL >2264-1316_1 YPSDHRR >2265-1316_1 APTRDQS >2267-1316_1 DYWPFFS >2268-1315_1 WSLSWFA >2271-1315_1 TQNVRLS >2273-1313_1 TSYVGIY >2275-1312_1 LILPTWG >2283-1306_1 WSLIPLY >2284-1305_1 FHNPTWN >2291-1301_1 FWGPSWM >2292-1299_1 HTAEAVR >2293-1299_1 SQDISAY >2299-1297_1 ADNPMWQ >2311-1290_1 EVYPPWM >2312-1289_1 DLKSSML >2322-1282_1 NFVDPSW >2326-1280_1 ANPNGHD >2334-1273_1 TLGHSRQ >2336-1270_1 FIGPSWD >2337-1270_1 SRDFVYG >2339-1269_1 YFTNPTW >2343-1264_1 NIDRDAM >2348-1261_1 YTPLWHM >2350-1259_1 VFWNPSW >2351-1259_1 WVQEWYD >2358-1255_1 SFFHPSW >2359-1254_1 FAYPTWW >2360-1254_1 FTPPPWQ >2366-1252_1 LGANLWW >2368-1251_1 EYWPIWT >2369-1250_1 HSFLIAQ >2370-1250_1 LSAREPY >2371-1250_1 HSLFFPS >2372-1249_1 FIIPSWG >2374-1249_1 MFTDPPW >2375-1248_1 TLGNLSL >2381-1245_1 YPRVAST >2382-1245_1 FMMPPWN >2383-1244_1 WWNPPWW >2387-1242_1 SFYYDWG >2390-1240_1 TYLPSWL >2395-1237_1 HGQDISA >2399-1234_1 YTKPMSM >2405-1233_1 FINPTWA >2429-1220_1 AFVVPSW >2431-1220_1 SFVDPSW >2432-1219_1 YVSPPWG >2434-1219_1 NYVHPSW >2440-1216_1 SSCRRGR >2446-1213_1 YNLPSWL >2447-1212_1 HFNEPTW >2448-1212_1 GNPQWQV >2451-1211_1 SVVSYSS >2455-1208_1 WNPFWYT >2460-1205_1 GKTDLGI >2462-1203_1 QVSNRFV >2468-1199_1 TPVGPVG >2470-1198_1 QFVSPPW >2476-1194_1 GPLWHLS >2479-1193_1 LWWYPSV >2482-1188_1 TPMQNTN >2483-1188_1 YPWFITE >2486-1187_1 TFWPVWE >2492-1184_1 SGGGRSL >2497-1182_1 NVALQSF >2501-1180_1 WYIQPSW >2505-1176_1 IWHSPPW >2508-1175_1 KQVTPPW >2512-1173_1 YPWAQMG >2519-1170_1 HYKSDRT >2521-1169_1 MPPWFAW >2523-1168_1 FSAYWNT >2524-1168_1 YNPFMDA >2528-1166_1 ASTVLDL >2529-1165_1 SSLGTRI >2537-1160_1 HWVMPSW >2540-1158_1 NVALQLL >2543-1157_1 FVLPSWI >2544-1156_1 SLIFPPW >2558-1150_1 FTSPSWA >2559-1148_1 FSCLLGG >2563-1148_1 FKFPTW* >2565-1147_1 VESLRMQ >2566-1147_1 TLINLRV >2570-1144_1 AWVSPSW >2574-1142_1 ASPMHVP >2579-1141_1 ASAPNPR >2580-1141_1 DFSWPTW >2581-1141_1 WWDPSWE >2582-1140_1 FVDPPWR >2584-1139_1 LTDH*TW >2586-1139_1 LHKGIHP >2590-1136_1 SIPLQTL >2592-1135_1 IMDLPAA >2595-1134_1 FQNPTWF >2602-1128_1 CVFGYCL >2603-1126_1 LLQPLYI >2604-1126_1 ALRMGPM >2606-1125_1 VATKHDY >2607-1125_1 YPKWEGI >2611-1124_1 WYMPSWM >2617-1122_1 EIFPTWA >2619-1120_1 WSWPSWS >2624-1118_1 YDPWAQY >2628-1116_1 TITRSAH >2633-1115_1 STPRNWA >2637-1114_1 TYWPVPD >2639-1113_1 INPQWQE | >166-4304_1 RVIAPSD >204-3575_1 WPVPYPL >259-2906_1 QWDDHW* >326-2221_1 QSPYPLN >366-2046_1 WPAREYF >385-1959_1 TLSGSRF >394-1905_1 TPQPYPL >436-1746_1 YETWEHR >442-1729_1 THSIFVY >471-1651_1 DWRNHWL >481-1612_1 AVSIVKR >504-1553_1 QWDDHWY >510-1534_1 RHEWLLH >543-1431_1 WLTPYPL >581-1355_1 AIPRLEE >608-1296_1 YHDAYPM >634-1249_1 YEGSIPH >646-1236_1 SPSPYPF >679-1179_1 VPSAYPL >689-1168_1 QANNTHM >698-1143_1 FWNTEML >702-1138_1 YNPWTHW >708-1131_1 NFRILSI >738-1088_1 THYRVNS >754-1059_1 KQPLHWY >759-1056_1 DLTPYPL >778-1031_1 DQPMHWF >819-979_1 FSWRMMT >823-976_1 DHVSWHY >836-963_1 QWEDHWQ >885-924_1 QDPYPMN >892-921_1 DHTSWHP >920-898_1 SGRIIGS >931-888_1 SHVSWHT >940-883_1 MHRMELR >964-861_1 GHEPYPL >969-858_1 DVPNWRT >977-845_1 MLTPYPL >982-844_1 YPNTDMT >984-842_1 AWPRHAA >986-842_1 VTITVQR >993-836_1 CLAGGRP >994-836_1 NPSAYPM >998-835_1 THVSWHT >1006-830_1 DMPSQWG >1028-809_1 LRQPATG >1036-805_1 DMPLWWT >1073-780_1 EHPSWYP >1075-777_1 GGLIIGM >1077-774_1 ASNANLR >1083-770_1  WMPMRDR >1092-766_1 YPPFTDP >1107-755_1 GKPCEHC >1113-751_1 EFRLPQL >1116-749_1 TPQPYPL >1117-746_1 FNVGVKP >1136-731_1 QCDDHW* >1140-728_1 NHTPYPL >1146-726_1 LSKFPTS >1156-724_1 TVAQATS >1159-722_1 YRPSMNS >1245-684_1 SWAIRIY >1252-678_1 NWLKVGT >1256-676_1 TAWSVMK >1292-660_1 TVEYITN >1301-656_1 TFVSMPP >1315-648_1 RLPMVEP >1330-642_1 NALTGSA >1334-640_1 HYKPVYP >1344-636_1 IPHWVQT >1367-626_1 RPEPYPL >1395-610_1 QWEDHC* >1397-610_1 GQRVVIS >1399-608_1 TPQAYPL >1401-606_1 THSPYPM >1427-593_1 AHLSIIF >1441-587_1 QSSKFLH >1458-579_1 MSMSQWR >1474-574_1 QAALNRM >1477-573_1 FRPPMMD >1493-567_1 IGVHHWN >1495-565_1 WGLHTFS >1500-564_1 LPGFSVS >1503-563_1 KCCATHL >1505-563_1 DQPMHWI >1515-559_1 MSHMDSP >1538-552_1 YILRAGD >1540-551_1 CMQAHAM >1548-547_1 DHLMHQR >1553-544_1 QWEDHWE >1573-539_1 MQTRLMA >1607-527_1 HTTKPSL >1611-527_1 TVTPYPM >1612-527_1 ACLMCLT >1613-526_1 HPFNQLQ >1620-523_1 WTIVGPI >1622-522_1 KMEPYPL >1630-521_1 MHSPYPL >1638-518_1 QLDPYPL >1642-517_1 HLTAVLT >1654-513_1 MDILAFH >1658-512_1 KPNIAIL >1663-509_1 QSYLYGW >1679-506_1 QLEPYPL >1706-498_1 AYPLYPT >1709-497_1 EQPVHWS >1711-497_1 GVQKASN >1719-494_1 MTFTIAA >1721-494_1 DSFRVWP >1738-487_1  HLLNGKP >1743-486_1 AHDPYPL >1747-484_1 DQPSHWM >1748-484_1 NAHTYGI >1750-483_1 HGLHQFN >1755-480_1 VMRPVTA >1756-480_1 YPEKSPP >1758-479_1 LTHATMR >1761-477_1 APHRLTQ >1762-477_1 NMSPYPM >1764-477_1 GYSLHHR >1772-476_1 ELKIIQP >1798-466_1 FRLPMTD >1810-463_1 MFTRGQE >1816-461_1 RPPMNDL >1817-460_1 *IDPYPL >1823-458_1 ITSSHVW >1825-458_1 LRLPMHD >1826-458_1 HFGPRQY >1840-454_1 GPLMPPY >1847-451_1 VGTIPPL >1852-450_1 THVSWHI >1856-449_1 TSSVSLM >1858-449_1 DRSAFSS >1865-448_1 GYCAEDT >1866-448_1 VLGPYPL >1867-448_1 FRLPMND >1872-446_1 TCSAKWC >1875-445_1 GPTRYPL >1876-445_1 HLGPEQP  >1885-442_1 ERDMRGI >1905-438_1 KDWPGRL >1924-433_1 QVTLSGH >1929-432_1 THWTRAD >1937-431_1 NKVIYYP >1959-427_1 LKTMPMI >1960-427_1 HLQWST* >1972-425_1 YGGFPMT >1981-424_1 VRSPFPM >1985-423_1 RPLPYPL >1986-423_1 HSLPHER >1987-423_1 YLSPYPL >1989-423_1 KATYIVL >1996-421_1 DRNMHIP >1997-421_1 ITKPEPN >2000-420_1 GYGKDWK >2008-418_1 MSYQIKR >2009-418_1 YTLSLSL >2015-417_1 NLEPYPL >2017-416_1 GMQPKPV >2022-414_1 WPGPYPL >2024-414_1 EHPSWAL >2060-406_1 ALTPYPL >2070-403_1 AHVPFHH >2073-402_1 DQTGGTN >2079-401_1 YARMLLS >2085-401_1 KMTPYPL >2089-399_1 TDWLVWE >2091-399_1 EAVPLRS >2093-399_1  TGSPSMA >2101-398_1 KQMPLYH >2105-398_1 SCPMCYW >2117-396_1 QEPYPLN >2119-396_1 KTSADMM >2126-394_1 GWSEHWS >2128-394_1 TDSRHVL >2131-393_1 DSWQKMR >2139-392_1 RPPMFDS >2141-392_1 HYKASLM >2151-390_1 HMTPYPM >2152-390_1 RIPMFDN >2163-386_1 MLMPYPL >2176-384_1 NQAKWVH >2184-383_1 SIPQASF >2191-381_1 WGLHTFR >2202-378_1 SLLMPLQ >2215-374_1 LSPTYLE >2216-374_1 RVPMNDG >2217-374_1 MTDSRAL >2220-373_1 WGVHQWR >2221-373_1 KFDGPMQ >2229-372_1 HLNANSK >2230-371_1 VYRSVEH >2235-371_1 HPFNRTS >2238-370_1 SF*TDHL >2245-369_1 VGMHTWN >2252-369_1 GVAHMTT >2257-368_1 DQPMHWL >2262-367_1 QWADHW*  >2264-367_1 YGSAFTM >2266-367_1 SPMPYPL >2268-366_1 EHFSLHP >2270-366_1 THSPYPM >2273-366_1 MRLPMHD >2280-365_1 NHGPYPL >2294-364_1 SLMPYPL >2303-363_1 GQPVHWF >2314-361_1 HPVPYPM >2315-361_1 FPP*KHV >2323-360_1 VLTPYPF >2329-359_1 VVSASWL >2342-358_1 SVSETGG >2362-354_1 QDPYPFS >2369-353_1 WRLPMSD >2375-352_1 ELGSALL >2380-352_1 TIGRYPV >2387-351_1 KLSPYPL >2400-349_1 GDQGPNP >2424-346_1 DQPRHWM >2426-346_1 MPDPYPL >2437-344_1 VLGWNMN >2453-342_1 LQPHHCY >2468-340_1 ASIFTTW >2471-340_1 SWSNVSR >2473-339_1 SSNLTDR >2478-339_1 QSKYPLD >2481-338_1 LPSLQVL >2485-338_1 LDRQALM >2489-338_1 NHMLSAK >2510-334_1 NQPYHWL >2533-331_1 YGLHTFH >2571-326_1 SADHRML >2575-326_1 WRTHTWA >2577-326_1 DQPWHWI >2578-326_1 HYSYRSI >2579-325_1 NTFAFNH >2592-324_1 QYPYPLD >2594-324_1 QWEYHW* >2595-324_1 MTPLDAR >2610-322_1 NYWTQHR >2620-321_1 TNATTEL >2632-319_1 TRAPMHD >2633-319_1 HPFNRSH >2641-318_1 QWEDHCY >2653-316_1 QLTPYPL >2654-316_1 LHPHHWY >2674-313_1 YTFPFAS >2680-312_1 QWRSHWN >2681-312_1 WPLPYPM >2687-312_1 YEGGSMG >2693-311_1 RVPMVDG >2707-310_1 DPTPYPL >2710-309_1 NAKTSHM >2711-309_1 TDGQLRR >2718-308_1 SLEPYPI >2719-308_1 WMPAMKG >2746-304_1 RIPMVDS >2748-304_1 NTALVPF  >2754-304_1 KVTNAQM >2763-302_1 GQPLHWA >2764-302_1 NPDPYPI >2772-301_1 NWPYMTW >2781-300_1 NTYKLSP >2785-300_1 DTLDALV >2795-299_1 YHYASMS >2800-298_1 TERHPRL >2811-296_1 KLDPYPL >2817-296_1 SLMPGRP >2823-295_1 SVLLSTK >2831-294_1 SALVALD >2832-294_1 LATPISD >2836-294_1 RLPFIDS >2841-294_1 SGSHKMS >2858-292_1 *TAYPLT >2861-291_1 YPGPYPL >2868-290_1 YLSPYPF >2869-290_1 TSI*TQT >2886-288_1 KPVSGHC >2899-287_1 VGGSLVG >2902-286_1 AAMHFNW >2909-286_1 AIGASDH >2915-285_1 YETWEHR >2918-285_1 GPTPYPI >2927-284_1 SGKVRPN >2928-284_1 STHTQPS >2929-284_1 NGLWHAD >2936-284_1 TLQPYPL >2939-284_1  YGLHSWR >2951-282_1 ATRPPWI >2959-281_1 WGVHQWS >2971-280_1 FNAPRPS >2977-279_1 LPMPYPL >2999-278_1 HTRMSPF >3006-277_1 QSPYPML >3010-277_1 RLPMIDA >3015-277_1 TLGLIKL >3018-276_1 HFRLHEP >3019-276_1 NGSYTWI >3025-276_1 RIPMDDP >3028-276_1 RLPMQDY >3032-275_1 YPHNLPP >3034-275_1 SWSPSGT >3035-275_1 RIPMNDP >3044-274_1 KPVHPSF >3046-274_1 HPHNQTQ >3051-273_1 TISDWTS >3060-273_1 HWSQLQA >3065-272_1 GFSGTVW >3068-272_1 HHIETAR >3082-271_1 WGTRIHA >3090-270_1 EAARILL >3098-269_1 QWEDHWH >3107-268_1 RPSTEPP >3112-268_1 KTLLQND >3113-268_1 VFSGVPL >3119-268_1 GANRETL >3127-267_1 SLSAYPF  >3135-266_1 LIAYDGL >3139-266_1 RVPFTDM >3146-265_1 SNLKMGY >3149-265_1 RLPFTDT >3166-264_1 YPAKFSN >3172-263_1 RTPMSDV >3184-262_1 VNREAID >3185-262_1 WSVTLDH >3187-262_1 ANVDPYS >3190-261_1 GPLPYPL >3191-261_1  VGGDYIM >3192-261_1 RPPMQDI >3207-260_1 WTPMHDP >3210-260_1 QWEDHL* >3223-259_1 SPFQSRL >3225-259_1 LIPAHSG >3226-259_1 TLTPYPL >3232-258_1 SPGLRSQ >3242-258_1 HLAYLMM >3257-256_1 AHKMAGN >3261-256_1 SGVKHDQ >3267-256_1 ELSAYPL >3284-254_1 TEWKYPL >3288-254_1 TPQPYPL >3289-254_1 ESMWIVT >3298-253_1 ADP*CQ* >3309-251_1 STTSPSK >3311-251_1 STVKTEG >3314-251_1 FATRITF >3316-251_1 RRVNH*P >3328-250_1 KLAHACI >3330-250_1 QNHDTLK >3344-248_1 QTPYPHN >3347-248_1 YNPWTHC >3350-248_1 NVMVALL >3354-248_1 NLSGWKW >3357-247_1 VGVHTWS >3360-247_1 SPVVGTE >3364-247_1 VMTPYPM >3377-246_1 SATPYPL >3382-245_1 HISKGMT >3390-245_1 TRVPMVD >3391-245_1 HPHNGNR >3407-243_1 HWEHHWR >3416-243_1 YWWTHRH >3421-242_1 THFRQGF >3425-242_1 QVSQSQP >3426-242_1 TVSPYPM >3433-241_1 HINPEAR >3437-240_1 VTYTRYT >3442-240_1 EQATLSP >3444-240_1 QWEDH** >3449-240_1 TQQKHDL >3452-239_1 NNELKIM >3453-239_1 AHPHRGG >3465-239_1 AWSMLWS >3474-238_1 EPSKYPL >3480-237_1 VVNQIFI >3481-237_1 WTPMQDP >3490-237_1 IYADIGF >3491-237_1 YTSMFTY >3496-236_1 AVRWTVP >3502-236_1 TQGYATS >3506-235_1 SGLYHFH >3507-235_1 TMPYLRI >3511-235_1 VQAGVWP >3519-235_1 GLMPYPL >3530-234_1 RIPMSDS >3531-234_1 WHWTDLK >3532-234_1  GSIWFSV >3535-233_1 VYHPTSV >3540-233_1 RDPVPIP >3543-233_1 EPGPYPL >3551-233_1 YRTPMID >3554-232_1 ASSYSMY >3562-232_1 WPSAYPM >3570-231_1 DQPRHWP >3571-231_1 ETIRNIG >3579-231_1 QWEDQWY >3581-230_1 GANNERW >3583-230_1 RFPMYDN >3587-230_1 SYWNEHR >3591-230_1 RPPMTEG >3592-230_1 RPPMTDF >3595-229_1 NWMEHWH >3596-229_1 GL*THPL >3599-229_1 RPPFMDG >3602-229_1 ALKVQKT >3627-227_1 SQFYTFW >3631-227_1 IGLYTFN >3636-226_1 FCTLSCI >3637-226_1 NDPAGHF >3638-226_1 H*NADLY >3641-226_1 NPLKFTS >3642-226_1 QREDHW* >3643-226_1 QVSWWMR >3648-225_1 YRSVTPL >3652-225_1 FHTAWHP >3655-225_1 FVANAPG  >3659-225_1 RHLVTHV >3668-224_1 SMDPYPL >3670-224_1 RLPFTDH >3679-223_1 LTTREML >3682-223_1 LTPTHIS >3684-223_1 HTLVTAR >3686-222_1 VIHY*LT >3689-222_1 FEC*RDE >3691-222_1 THTPYPM >3696-222_1 KGMHHFN >3698-222_1 SISYFIT >3704-222_1 RQKQPYG >3712-221_1 GRWDLHS >3713-221_1 GHVSWHK >3714-221_1 SPEKYPL >3718-221_1 TLTPYPV >3722-220_1 LHVAWHA >3725-220_1 *ELKNGT >3726-220_1 TVSMRPP >3733-220_1 PRD*SA* >3736-220_1 SPPLAL* >3741-220_1 SSNASLH >3757-219_1 RIPMFES >3759-219_1 HSQVTQQ >3761-219_1 YPPGNSW >3775-218_1 WPTRYPL >3781-218_1 KLTATPM >3785-217_1 *ISPYPL >3808-216_1 GVERPIR >3810-216_1  WIYTDLK >3823-215_1 TSVGSPD >3824-215_1 RR*QMYP >3825-215_1 FPWKGHV >3830-214_1 DTRFVQT >3832-214_1 FLTPYPI >3848-213_1 LRVPMHD >3858-212_1 FHEPYPF >3862-212_1 YTKPMSM >3865-212_1 LGLHTWS >3869-212_1 TPQNTRI >3881-211_1 VLEAYPM >3883-211_1 HPRAATD >3889-210_1 HPHNRPV >3892-210_1 FETYYSW >3897-210_1 KSMVPNV >3899-210_1 VRHVPHA >3909-209_1 QEYTWMN >3914-209_1 TFPLMRP >3921-209_1 *IPYPLS >3926-208_1 VPVHKYD >3927-208_1 VNLRYYI >3937-208_1 HPFNRTG >3939-208_1 SHVSWHE >3941-208_1 NTTNSHG >3956-207_1 SPTRYPL >3959-207_1 APDPYPL >3969-206_1 NGSAKDP >3972-205_1 SFGVRSV >3982-205_1 YDLIRRE >3983-205_1 NALFQTN >3987-205_1 VRWPMHD >3991-205_1 SFSQGRT >3999-204_1 MNTIELK >4000-204_1 DAGMGQR >4014-204_1 HHT*LPK >4017-204_1 GVSLVVL >4020-203_1 VSSVYFP >4027-203_1 WGAVHTT >4030-203_1 TEPSHKN >4036-202_1 ESKLNTS >4038-202_1 RPEAYPL >4044-202_1 VPSDEHM >4050-202_1 ETDKDSR >4052-202_1 WGLHNFA >4059-201_1 LQTHHWY >4060-201_1 HNRLHEN >4063-201_1 RVPMSDY >4074-200_1 TRFMQST >4077-200_1 SLTYPNH >4078-200_1 TWNSHWN >4085-200_1 HTYPHLL >4109-198_1 NAHNDKS >4117-198_1 MHVTLFA >4119-197_1 TGATIFK >4123-197_1 KPSPYPL | >101-10104_1 VFIPYGH >181-5658_1 KVMQLHI >397-2768_1 QMQWAVT >431-2573_1 YRGISVN >461-2449_1 AYLFNPM >502-2225_1 HAPIGNN >563-2049_1 RDLATLQ >574-1993_1 TVNNRMY >585-1953_1 GVWMLPK >643-1824_1 LVSSYNF >645-1813_1 TSIMFRY >672-1742_1 SVSTNSM >681-1724_1 ETRT*TL >683-1714_1 QPGRIVQ >712-1650_1 GIGRVAL >713-1649_1 LVALRVG >753-1558_1 ASGSLCC >758-1552_1 VVPCAGL >763-1539_1 TYTNPGF >767-1533_1 ETGSNTI >776-1520_1 VMDLRIL >791-1500_1 ISLRIEM >826-1450_1 MPFWLAR >840-1424_1 MPYDRSR >875-1363_1 RIHVDLF >878-1361_1 TSIGVFS >915-1301_1 WNSVSRL >933-1283_1 FFWMTRI >975-1234_1 VSPVRQL >987-1223_1 MYQINVT >1016-1174_1 SGEQYRI >1021-1170_1 VPMQTRL >1030-1153_1 SHTNPKS >1052-1127_1 NILANDR >1067-1113_1 ISTHYYL >1081-1097_1 WTVVQTL >1096-1083_1 H*EISVL >1181-1021_1 YSYVHTV >1190-1015_1 HYSATAL >1191-1014_1 QPSYWSR >1201-1005_1 YVQGSRL >1205-1001_1 WPSFVPK >1210-997_1 TFAGYRV >1211-996_1 SISHRMY >1223-986_1 HVRANVP >1227-983_1 WNLGGGS >1231-980_1 ASKLSTG >1238-976_1 YQPVTYR >1261-960_1 QPDSNVA >1272-955_1 VIHGNPP >1276-954_1 SF*TDHL >1279-952_1 QQHHVHL >1300-939_1 VSPRAGM >1322-928_1 DRNPMSF >1334-920_1 DPAT*TV >1348-914_1 GSPQVKK >1403-878_1 RVQDLPH >1413-874_1 SFRLGSH >1415-873_1 TVHAAYM >1430-865_1 WGQLGIR >1466-848_1 HISKGMT >1477-843_1 AVQFL*T >1493-838_1 GVNIRGI >1505-830_1 GQRVVIS >1515-826_1 MVHDMLG >1536-817_1 GNGPLHW >1537-816_1 VGKMHAN >1550-807_1 LAAKSGT >1552-806_1 GTSPDVW >1555-802_1 HTLVTAR >1563-799_1 VSWSSSS >1568-798_1 ITWSEHP >1586-789_1 AYFNLLR >1592-786_1 ASHAVGV >1600-780_1 TEWLRFD >1615-770_1 SLKHPMY >1625-763_1 MSWKLLQ >1627-762_1 LNIKLTL >1654-747_1 QTGLSSS >1675-737_1 AFHVTRM >1677-736_1 AYPKADS >1680-735_1 VGTTVVK >1694-730_1 TGSLFLT >1715-722_1 IARFSAN >1730-716_1 HTPVLSN >1737-713_1 VSYGGRT >1739-711_1 SIYSWAV >1743-708_1 FRFSCRW >1750-703_1 NGLVYRE >1752-702_1 RYLDVWA >1756-701_1 EMYFTKQ >1763-698_1 ISLTEHP >1773-694_1 AQMPVNV >1778-691_1 YPHPRWT >1790-689_1 FSSLGSK >1791-689_1 WSTLTPP >1795-688_1 SAPDDSF >1796-688_1 TWIRSAL >1797-688_1 MLQTNYD >1810-682_1 DRHFNYL >1816-681_1 QLSNCCQ >1824-678_1 GSTSPIV >1843-671_1 LERMPNP >1847-667_1 MWRIDFR >1849-666_1 SQSTVLQ >1857-664_1 HNPGTHK >1871-660_1 SGLDLWT >1893-654_1 QTSGSKL >1906-649_1 QSQIPNS >1921-645_1 SLPLVYS >1934-641_1 HSWIMHT >1938-640_1 WCRGLVW >1947-637_1 TFTSYLT >1949-637_1 AIMHHMV >1957-633_1 ASLDRYN >1966-631_1 MIKYRVA >1967-631_1 SMLIPHP >1970-630_1 SLYP*IG >1981-625_1 VMQGVLF >1998-619_1 NHVS*VS >2000-619_1 TPHWYVK >2006-616_1 TEQYTLN >2010-615_1 QMRPQHT >2012-614_1 KLYQREP >2017-614_1 GSNSARS >2035-606_1 NATMSAS >2043-604_1 IYPKVGH >2052-602_1 STYKSML >2069-597_1 MSRGAFI >2075-596_1 IWFTFQD >2085-593_1 APGGVVR >2093-591_1 ERVLPRV >2100-589_1 VPLWYVK >2115-585_1 ATRPLNH >2117-584_1 EQQTIRL  >2119-583_1 TWS*AQT >2132-580_1 QLWYKIN >2137-578_1 FPGVQTQ >2139-578_1 VLFPDIT >2145-577_1 TNKTFQL >2146-577_1 ALFSNGR >2153-575_1 EASTKLT >2157-574_1 AIRAPWS >2164-573_1 IGLSEHF >2179-568_1 SVPYKLL >2198-563_1 AAPHPAT >2226-555_1 YRITIPM >2238-552_1 YVELIFV >2261-546_1 TPHWYVR >2268-545_1 NTSTYLM >2270-544_1 TVLSAAL >2282-542_1 TWPEFTK >2286-541_1 YGFTSNR >2299-539_1 ELQIVPS >2308-537_1 LT*KAQY >2313-535_1 AKVDPED >2314-534_1 YPHPRWN >2316-534_1 NFDT*AL >2332-531_1 VSLTYHI >2340-529_1 GVAHMTT >2347-526_1 NAPWWGS >2352-525_1 SLTEKNW >2355-524_1 VANTHHG >2373-520_1 ECRPCAD >2381-518_1 NIVSNSS >2387-516_1 DPLLNGV >2390-516_1 YMTGSKP >2393-515_1 *TWRGAV >2397-514_1 VLYSEHP >2400-513_1 KIPLTSK >2401-513_1 SVFSFRL >2423-509_1 SSMRQMH >2432-508_1 ALVFESY >2441-507_1 WPTFIPR >2442-507_1 LDPHRWM >2452-505_1 TYTGFVS >2456-504_1 EFAISST >2467-502_1 WKSGLTY >2472-501_1 GLIVSPM >2487-499_1 NTTTLES >2497-498_1 FQRSTAH >2503-496_1 SHQDNLL >2507-495_1 HQRPFVP >2509-494_1 FMILPGN >2511-494_1 YEGSIPH >2516-493_1 WRHVP*T >2527-490_1 LKLLDSY >2528-490_1 QLAYSTR >2529-490_1 GVHYTSA >2530-490_1 IHMSPPL >2548-486_1 HVFPCCH >2549-486_1 TYPERSA >2559-484_1 IPRWYVK >2565-483_1 *TSHKYL >2569-482_1 LARITAE >2586-480_1 VFAKANP >2587-480_1 HVDGSLR >2610-477_1 YRFVNFQ >2621-475_1 QWRKTES >2624-474_1 MAFTNAM >2663-467_1 SFYLPIR >2664-467_1 LTCLHCN >2673-466_1 EHPTTQI >2684-464_1 MGAMARV >2697-460_1 AVMDKRN >2699-459_1 DLFARSH >2701-459_1 TLGTPWS >2706-458_1 SQVAMIR >2709-457_1 GSSNLNL >2719-455_1 EPLSIFS >2742-452_1 YYPPAHR >2743-452_1 NAFKMVR >2744-452_1 YQRPFNP >2748-451_1 VSQMRSP >2772-447_1 GQMPFRP >2777-446_1 SPPLGSS >2781-446_1 IVDTTWH >2783-445_1 TVVMYPG >2784-445_1 SPDPYLR >2791-445_1 QVYDRSK  >2811-441_1 QGLSRPH >2815-440_1 SLFPMSL >2818-440_1 DTPLPPW >2826-439_1 WMTDRVV >2829-437_1 KPPMSKY >2833-436_1 SQGMNLR >2838-435_1 SHPWTSG >2841-434_1 WTTTWMY >2844-434_1 MFSTATA >2857-432_1 WMTPRWL >2866-430_1 LPTVRNS >2871-429_1 TPKWFVK >2879-427_1 QHYDRSR >2880-427_1 HWYDRDR >2904-423_1 HFFTLTI >2909-423_1 THLLIYM >2911-422_1 YPWSEQR >2927-420_1 ICWACSP >2928-420_1 TGSSWAP >2930-420_1 HLQTSYT >2938-419_1 GKLTNVR >2943-418_1 VGFYEHP >2950-418_1 RIPIH*N >2958-417_1 VLYTKNL >2960-417_1 DGGYWHV >2961-416_1 AISLTPR >2962-416_1 SLPT*GS >2969-416_1 RAPVNSV >2973-414_1 QEYNRER >3003-411_1 YPNTDMT >3019-408_1 LDNALDR >3020-408_1 DGKQVSI >3027-407_1 YVRLAVT >3039-405_1 NTQHVTS >3040-405_1 YCYWCNP >3046-404_1 SLEDETL >3048-404_1 HEHELKS >3060-401_1 GYSLHHR >3070-400_1 AADNMRS >3074-400_1 SYLLPIA >3079-399_1 IGPASEA >3083-399_1 SWSYYSR >3088-397_1 WMYYPYE >3091-397_1 WGTLGLR >3100-395_1 RPAITTM >3103-395_1 GDKTSPF >3108-395_1 GFGENSG >3115-394_1 ARTVFSY >3125-392_1 REVMHWP >3127-392_1 IQYTEHP >3133-391_1 WTSHHYY >3139-390_1 HAMTEAP >3140-390_1 VTSYFST >3141-390_1 MVPRIHP >3155-388_1 GMTLATL >3156-388_1 TTKFIAK >3161-388_1 KWMDHQW >3170-386_1 AGAQDSM >3182-385_1 LPSPIIT >3186-384_1 EPTLSKP >3187-384_1 EQEYYPF >3188-384_1 FQAPFRP >3191-383_1 TDWLVWE >3196-382_1 RTDYPYV >3205-382_1 DPWEWSF >3211-381_1 TQKPFRP >3212-381_1 FPPNLYT >3222-380_1 TDTGANW >3234-379_1 YMASTHT >3235-379_1 FHEHMTA >3243-378_1 SLGRIVT >3254-377_1 NIGLLAN >3263-376_1 TSSMTYL >3270-375_1 TAIWQAS >3275-374_1 GLSVRPT >3279-374_1 GYWNLAY >3281-374_1 GVQKASN >3295-372_1 AVFTRQP >3297-371_1 SYNGLAS >3308-369_1 SPMYWGN >3312-369_1 GHL*NAL >3330-367_1 NPFTNSA >3333-367_1 HGPNTST >3339-366_1 MTLSRVP >3352-364_1 TFTATFS >3353-364_1 GSIITHH >3354-364_1 VVTQRHL >3356-363_1 STSELYG >3365-362_1 DQRPFKP >3367-362_1 ELGAYKL >3388-359_1 VALWPHP >3389-359_1 IVYLEHP >3403-358_1 ALNISGL >3422-356_1 MPDAVNQ >3425-355_1 GAQLYDN >3430-354_1 ELTRFAK >3449-352_1 YNGKALS >3458-350_1 LGSLVRG >3464-350_1 IVMPQEW >3466-349_1 QFKTSNT >3470-349_1 HQAFPMK >3479-348_1 SGSSGHR >3483-348_1 TLPSYRN >3489-347_1 YYQAELP >3508-345_1 TWAVNTN >3513-345_1 FKQNLVM >3517-344_1 FQVPFRV >3525-343_1 ASRLTNL >3529-342_1 WHQTARE >3534-342_1 AGQLKHE >3549-339_1 NILWKSS >3551-339_1 NPRTWLD >3554-339_1 DTKHNLL >3567-337_1 EYSLVAS >3573-337_1 STEAWSA >3578-336_1 TSIFTRF >3587-335_1 QLFTTYV >3596-334_1 GIGGTPN >3597-334_1 WPMSVPR >3598-333_1 AFWPTFV >3610-331_1 ALLGKSP >3622-330_1 SYPREGY >3624-330_1 GSENNHH >3629-329_1 VSIHLSL >3631-328_1 VTLKHGG >3636-328_1 ATPATYY >3643-327_1 SLKLAFR >3655-326_1 SQGHMMI >3659-326_1 SKHSAML >3660-326_1 DYTRERW >3667-325_1 WSTSISG >3668-325_1 VERWYLQ >3679-324_1 NEDWFAI >3680-323_1 VQIMWPW >3684-323_1 WGTRIHA >3686-323_1 FMDAHIA >3688-322_1 STFYPPM >3690-322_1 KLNYAGM >3701-320_1 APTATIF >3703-320_1 TLGMDAE >3704-320_1 MTPEGPL >3708-320_1 GSTRTHD >3714-319_1 SFLLPVF >3721-319_1 LGSPLPI >3760-315_1 WSVTLDH >3768-314_1 YGLASTA >3769-313_1 NLFGFPI >3774-313_1 LSNQPPS >3780-313_1 QFNVYIV >3789-312_1 FLWTSTS >3792-312_1 DRALFFP >3795-312_1 VDITRMR >3799-312_1 ALLPSNY >3803-312_1 YPILRNA >3805-311_1 STCSLLR >3812-310_1 TLSLPSW >3820-309_1 TLYVRTK >3838-308_1 QHFPFAF >3856-307_1 SFLLPVH >3867-306_1 TRPLAHM >3883-304_1 ESLGEEP >3884-304_1 TKTHSNL >3887-304_1 SYVKIEL >3896-304_1 WNSIEST >3911-302_1 KTHPSCP >3915-302_1 VYVGPDI >3920-302_1 HDTRKPL >3931-301_1 VSNPFHL >3953-300_1 VVPCAGL >3957-300_1 QLHAGQY >3960-299_1 ASWTDRY >3961-299_1 FLFLMVM >3963-299_1 VGMRHPA >3964-299_1 VVERTIR >3974-298_1 A*GYPDT >3984-298_1 HT*DKPK >3990-297_1 YSANHGA >3992-297_1 LNARDYT >3994-297_1 GKQYVMA >4017-295_1 *MHYHHL >4020-295_1 TFKCLHN >4025-294_1 ILHELNP >4026-294_1 TRVTQYL >4033-294_1 HPGYRVH >4042-293_1 ITVHEHP >4048-292_1 TLMDLFM >4050-292_1 AGANHYL >4059-291_1 ALGITQV >4066-291_1 NGAYLRC >4071-290_1 EQTPFRV >4081-289_1 YANFTPS >4086-289_1 VGSSF*N >4090-288_1 GKLYIKS >4092-288_1 YRLAPKM >4095-288_1 AVHGHLI >4119-285_1 VERWYLM >4123-285_1 T*MPFHP >4131-284_1 TQRQLTI >4133-284_1 YPPL*AW >4144-283_1 SFWLPVL >4145-283_1 WPFASAF >4151-282_1 MGEYHTR >4155-282_1 GTSFSGH >4162-281_1 LMVTVCP >4165-281_1 TYKDLVQ >4172-280_1 RCASEIP >4173-280_1 NISDYGR >4182-279_1 IDTEYKL >4184-279_1 DAQTLTR >4187-279_1 SWWPLFP >4188-279_1 YTPPKAY >4189-279_1 ERNVQDR >4196-278_1 WLNKLPM >4203-278_1 GVANARF >4205-277_1 FSGHPAT >4215-277_1 RLLDGLL >4217-276_1 SSTVNLR >4219-276_1 NWLPMTF >4222-276_1 DRALHWP >4224-276_1 VNPWMPH >4226-275_1 HQWMSLT >4227-275_1 TRTLWTT >4228-275_1 SSGTRHD >4230-275_1 DSANPVN >4231-275_1 FLPTPQV >4239-274_1 T*MPFTP >4246-274_1 IASSVTQ >4247-273_1 MPP*KLV >4256-273_1 KPWTLLG >4264-272_1 PLILRLL >4265-272_1 VPRADVL >4285-271_1 EPMDRYI >4288-271_1 GPPWGSA >4301-269_1 KSPSNIR >4303-269_1 SFLLPVL >4306-269_1 GLRDICC >4316-268_1 VEWCSSQ >4327-268_1 VYIIGAV >4336-267_1 LMQQRQP >4338-267_1 SVDLTLK >4342-267_1 QAMPLND >4349-266_1 WDIFMTY >4361-265_1 VGM*VLF >4367-265_1 NTLKAYN >4368-265_1 KSLEDGS >4369-265_1 WDITQER >4372-265_1 WPAIRGQ >4374-264_1 YSYSRNT >4375-264_1 ASSARHY >4382-264_1 MLAGAPT >4383-264_1 ATWLDRY >4385-264_1 HHQRQIS >4390-263_1 ANSYYRY >4391-263_1 FHMETGM >4400-263_1 VTYDRER >4417-261_1 TVTYLTV >4428-261_1 NWDHLTT >4431-260_1 LPNRLTQ >4432-260_1 NMRSYAN >4433-260_1 HCTHCMP >4438-260_1 GVERPIR >4441-259_1 YTLELMF >4445-259_1 RLKANQT >4457-259_1 MIFSEDD >4458-259_1 YIPDNGD >4460-259_1 TTYKSTH >4463-258_1 VITVVFG >4470-258_1 LDI*IAI >4472-258_1 SQSSNHL >4474-258_1 LTGTYSI >4481-257_1 QATVVVP >4483-257_1 MNWNLVV >4485-257_1 YWHPRWH >4498-256_1 YSEMHIE >4502-256_1 VHEHRQY >4507-256_1 DPRQQEP >4510-255_1 ALYDRSK >4519-255_1 TFWPDHP >4524-254_1 SSARPAT >4527-254_1 YDRDKGW |
| Number of peptides with matches to PAP | 48 | 127 | 74 | 32 |
